# A μ‐Phosphido Diiron Dumbbell in Multiple Oxidation States

**DOI:** 10.1002/anie.201908213

**Published:** 2019-08-23

**Authors:** Munmun Ghosh, Hanna H. Cramer, Sebastian Dechert, Serhiy Demeshko, Michael John, Max M. Hansmann, Shengfa Ye, Franc Meyer

**Affiliations:** ^1^ Institut für Anorganische Chemie Georg-August-Universität Göttingen Tammannstrasse 4 37077 Göttingen Germany; ^2^ Max-Planck Institut für Chemische Energiekonversion Stiftstrasse 34–36 45470 Mülheim an der Ruhr Germany; ^3^ Institut für Organische und Biomolekulare Chemie Georg-August-Universität Göttingen Tammannstrasse 2 37077 Göttingen Germany; ^4^ Max-Planck Institut für Kohlenforschung Stiftstrasse 34–36 45470 Mülheim an der Ruhr Germany

**Keywords:** electronic structure, iron complexes, N-heterocyclic carbenes, phosphido complexes, redox series

## Abstract

The reaction of the ferrous complex [LFe(NCMe)_2_](OTf)_2_ (**1**), which contains a macrocyclic tetracarbene as ligand (L), with Na(OCP) generates the OCP^−^‐ligated complex [LFe(PCO)(CO)]OTf (**2**) together with the dinuclear μ‐phosphido complex [(LFe)_2_P](OTf)_3_ (**3**), which features an unprecedented linear Fe‐(μ‐P)‐Fe motif and a “naked” P‐atom bridge that appears at δ=+1480 ppm in the ^31^P NMR spectrum. **3** exhibits rich redox chemistry, and both the singly and doubly oxidized species **4** and **5** could be isolated and fully characterized. X‐ray crystallography, spectroscopic studies, in combination with DFT computations provide a comprehensive electronic structure description and show that the Fe‐(μ‐P)‐Fe core is highly covalent and structurally invariant over the series of oxidation states that are formally described as ranging from Fe^III^Fe^III^ to Fe^IV^Fe^IV^. **3**–**5** now add a higher homologue set of complexes to the many systems with Fe‐(μ‐O)‐Fe and Fe‐(μ‐N)‐Fe core structures that are prominent in bioinorganic chemistry and catalysis.

## Graphical abstract

**The naked truth**: Using a macrocyclic tetracarbene ligand scaffold and Na(OCP) as a P‐atom transfer reagent has allowed a diiron complex to be isolated with a “naked” P bridge. The complex exhibits rich redox chemistry, and both the singly and doubly oxidized species could be isolated and fully characterized. The unprecedented linear Fe‐(μ‐P)‐Fe core is highly covalent and is structurally invariant over three oxidation states.

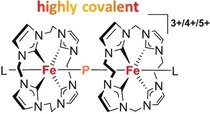

Stimulated by the great interest in reactive metal‐oxido and metal‐nitrido intermediates, substantial efforts have been devoted to isolating transition metal phosphide congeners with a single‐atom P_1_ unit. Following initial breakthrough reports on molecular compounds with a formal M≡P bond,^1^ a few complexes featuring a terminal or bridging “naked” phosphido ligand have now been isolated, but almost exclusively for early and mid‐transition metals such as Mo and W.^2^ More recently, μ‐phosphido linkages have also been structurally authenticated with actinide elements.^3^ In contrast, hardly any P_1_‐ligated complexes have so far been isolated for the most abundant transition metal Fe, even though related oxido‐4 and nitrido‐iron^5^ species are particularly prominent owing to their relevance to bioinorganic chemistry and catalysis.

More generally, molecular complexes with Fe_*x*_P_*y*_ cores have remained rare so far. Relevant examples comprise the dinuclear complex **A** (Figure 1) with bent μ‐P linkages and a rhombic Fe_2_P_2_ four‐membered ring^6^ as well as a number of systems with larger P_*y*_ units derived from white phosphorus (P_4_) that feature, for example, [Fe_2_(μ‐η^2^:η^2^‐P_2_)_2_], [Fe_2_(μ‐η^4^:η^4^‐P_4_)], or [Fe_4_(μ_4_‐η^2^:η^2^:η^2^:η^2^‐P_8_)] cores.^7^ Driess, Grützmacher, and co‐workers recently reported the synthesis of a mixed‐valent (Fe^II^/Fe^III^) diiron complex with an asymmetric cyclo‐P_3_ ligand (**B**) that forms as a product from the reaction of the corresponding β‐diketiminato iron(II) chlorido complex and Na(OCP).^8^ The phosphaethynolate anion (OCP^−^)^9^ is emerging as a suitable synthon for transferring P anions. Since practical syntheses of Na(OCP) and related phosphaethynolate salts have been developed,^10^ this strategy is now being successfully exploited for the preparation of various p‐, d‐, and f‐block metal complexes.^11^

**Figure 1:**
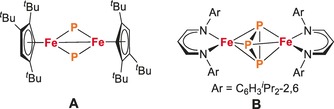
Selected Fe‐P_*y*_‐Fe complexes reported in the literature.^6^, ^8^

Here we report the reaction of Na(OCP) with the tetracarbene‐based iron(II) complex [LFe(NCMe)_2_](OTf)_2_ (**1**; Scheme 1)^12^ and the characterization of the first examples of Fe‐P‐Fe complexes featuring a single‐atom “naked” phosphorus bridge between two iron atoms. This now complements the well‐known μ‐oxido (Fe‐O‐Fe) and μ‐nitrido (Fe‐N‐Fe) diiron motifs, which hold prominent places in bioinorganic coordination chemistry and beyond.^4^, ^5^ Macrocyclic tetracarbene ligands such as L have previously been shown to serve as a rugged scaffold even under harsh reaction conditions and to support iron complexes in a variety of oxidation states,^12^, ^13^, ^14^, ^15^, ^16^ for example, the only organometallic oxoiron(IV) complex [LFe(O)(NCMe)](OTf)_2_.^12^, ^15^

**Scheme 1:**
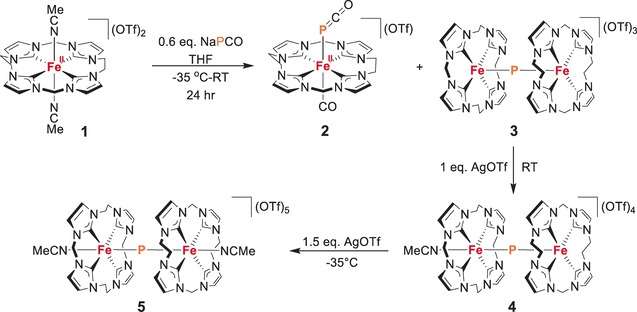
Overview of the reactions studied and compounds characterized in this work. OTf=trifluoromethanesulfonate.

The addition of Na(OCP)(dioxane)_2.5_ to a suspension of **1** in THF at −35 °C resulted in the immediate formation of a green precipitate that was characterized as the μ‐phosphido diiron complex [(LFe)_2_P](OTf)_3_ (**3**; Scheme 1). From the remaining yellow THF solution, single crystals of the ferrous complex [LFe(PCO)(CO)]OTf (**2**) featuring *trans*‐positioned OCP^−^ and CO ligands could be obtained; the molecular structure of the cation of **2** determined by X‐ray diffraction is shown in Figure 2 (Figure S57). Metal complexes with intact P‐bound OCP^−^ are quite rare, especially for 3d transition‐metal complexes.^17^, ^18^

**Figure 2:**
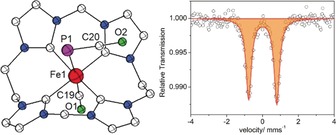
Molecular structure of the cationic part (left) and zero‐field ^57^Fe Mössbauer spectrum of **2** recorded at 80 K (right).

The P‐C‐O and P‐Fe‐C angles in **2** are close to 180° (178° and 175°), while the Fe‐P‐C angle is 95°; Fe−P and Fe−C(O) bond lengths are around 2.50 and 1.74 Å, respectively. Note that the Fe−P bond is much longer (ca. 0.5 Å) compared to those in **3** and **5**. As expected, the Fe−C(O) distance is much shorter than the Fe−C^NHC^ bonds of all the structurally characterized complexes in this study (1.74 versus 1.95–2.05 Å).

The diamagnetic character of **2** suggests a low‐spin d^6^ configuration, in line with other six‐coordinate ferrous complexes of the tetracarbene macrocycle L.^12^, ^14^ The ^57^Fe Mössbauer spectrum of **2** shows a quadrupole doublet with an isomer shift (IS) *δ*=0.05 mm s^−1^ and a quadruple splitting (QS) Δ*E*_Q_=1.57 mm s^−1^ (Figure 2, right), which confirms the *S=*0 spin state. The observed IS is remarkably lower than that of the starting low‐spin iron(II) complex **1** (*δ*=0.23 mm s^−1^, Δ*E*_Q_=2.10 mm s^−1^),^12^ which reflects the effect of the different π‐backbonding abilities of the axial ligands.

The ^31^P NMR spectrum of a solution of complex **2** in CD_3_CN at 238 K (also at 298 K) shows a signal at *δ*=−412 ppm (Figure S1), slightly upfield‐shifted from the signal of Na(OCP) in the same solvent (*δ*=−391 ppm). The IR spectrum of **2** (Figure S37) shows a band at $\tilde{\nu }$ =1905 cm^−1^, which is assigned to the CO stretch, and a band at $\tilde{\nu }$ =1832 cm^−1^, which is assigned to the asymmetric PCO stretching vibration; the latter would suggest a more cumulenic nature of the PCO unit compared to Na(OCP) (1755 cm^−1^).10a The ^1^H NMR spectrum reflects the apparent *C*_2*v*_ symmetry of **2** and reveals pronounced coupling between the P atom and the macrocycle methylene protons that are directed towards the OCP^−^ ligand (*J*_PH_=7.9 Hz, see Figures S2–S8). In the ^13^C NMR spectrum, doublets arising from coupling to ^31^P are observed for the adjacent C atom of the OCP^−^ ligand (at *δ*=168.9 ppm, *J*_PC_=94.4 Hz), and also for the carbene‐C atom (at *δ*=186.3 ppm, *J*_PC_=4.5 Hz) and the *trans*‐CO ligand (at *δ*=222.2 ppm, *J*_PC_=9.0 Hz).

Optimizing the synthetic conditions according to the reaction stoichiometry shown in Scheme 1, namely by using about 0.6 equivalents of Na(OCP)(dioxane)_2.5_ and conducting the reaction at −35 °C, delivers the two products **2** and **3** in approximately 60 % yield (see the Supporting Information for details). The diffusion of diethyl ether into a solution of **3** in MeCN afforded single crystals suitable for X‐ray diffraction. The molecular structure of the cation (Figure 3, top left) shows two {LFe} entities bridged by a single “naked” P atom. The two five‐coordinate metal ions are found in a square‐pyramidal {C_4_P} environment and the central Fe‐(μ‐P)‐Fe core is almost linear (angle Fe‐P‐Fe 178.3°) with Fe‐P bond lengths of 1.993(2) and 1.998(2) Å (Figure S58). These overall structural features are reminiscent of the related oxido‐bridged [(LFe)_2_(μ‐O)](OTf)_4_.^12^ The Mössbauer spectrum of **3** shows a quadruple doublet with parameters *δ*=0.01 mm s^−1^ and Δ*E*_Q_=1.94 mm s^−1^, quite similar to those of [(LFe)_2_(μ‐O)](OTf)_4_ (*δ*=0.04 mm s^−1^ and Δ*E*_Q_=2.56 mm s^−1^)^12^ and indicative of ferric complexes of the tetracarbene macrocycle L.^14^ Although μ‐oxido diiron(III)^4^ complexes are abundant and a number of μ‐phosphido complexes have been reported, mainly for 4d and 5d metals,1a, 3a, 3c, 19 the Fe‐(μ‐P)‐Fe core with a “naked” bridging phosphido ligand is unprecedented.

**Figure 3:**
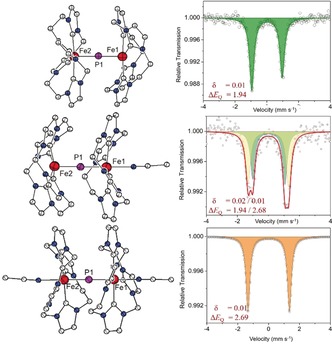
Molecular structures of the cations of **3** (top), **4** (middle), and **5** (bottom) as well as ^57^Fe Mössbauer spectra of the three compounds recorded at 80 K (right). The IS and QS values (in mm s^−1^) are given in the respective figures.

The ESI(+) mass spectrum of a solution of **3** in MeCN shows a dominant signal for the fragment [LFe]^2+^ at *m*/*z*=202.0 as well as signals at *m*/*z*=279.5 and 434.9, which are assigned to the ions [LFePFeL]^3+^ and [LFeP]^+^, respectively. This finding suggests that **3** might disproportionate into ferrous [LFe]^2+^ and the formal ferryl species [LFeP]^+^, at least under the ESI‐MS conditions (Figure S47). The potential existence of a terminal iron phosphido species [LFeP]^+^ is an exciting perspective. It should be noted that a disproportionation equilibrium has recently been evidenced for diferric [LFe^III^(μ‐O)Fe^III^L]^4+^ in MeCN to give **1** and [(MeCN)LFe^IV^=O]^2+^ (although the equilibrium lies far on the side of [LFe^III^(μ‐O)Fe^III^L]^4+^; *K*_eq_=9.7×10^−7^ m).13e

Complex **3** is diamagnetic even at room temperature (for SQUID data in the range from 2 to 295 K, see Figure S48), indicative of an *S=*0 ground state and extremely strong antiferromagnetic coupling of the two ferric ions (|2*J*|≥1200 cm^−1^), at least as strong as in [(LFe)_2_(μ‐O)](OTf)_4_.^12^ Accordingly, the ^1^H NMR spectra of **3** in CD_3_CN show resonances in the range *δ*=3.8–7.4 ppm. However, some signals are broad at room temperature but sharpen upon cooling to 238 K, thus suggesting conformational dynamics of the macrocyclic ligand scaffolds. DOSY in combination with 2D NMR experiments (NOESY, COSY, EXSY, HSQC, HMBC; see Figures S11–S25) at 238–298 K identifies three isomers of the trication [(LFe)_2_P]^3+^ that differ by the mutual rotation of the two {LFe} caps and by the conformations of the seven‐membered chelate rings, and that interconvert with rate constants between 0.1 and 0.5 s^−1^ at 298 K (derived from ^1^H EXSY spectra, Figures S16–S18; see the Supporting Information for a more detailed discussion). This is reminiscent of the isomerism observed for a derivative of [(LFe)_2_(μ‐O)]^4+^ with octamethylated tetracarbene ligands.^14^ In the solid state, the two macrocycles L in **3** are rotated by 5° with respect to each other and adopt saddle‐shaped conformations where the ‐CH_2_‐ linkages are oriented towards the other {LFe} cap, while the ‐CH_2_CH_2_‐ linkages are directed outwards, giving an approximate (noncrystallographic) *D*_2*d*_ symmetry to the complex. Dissolving fresh crystalline material of **3** at 238 K followed by immediate recording of an NMR spectrum already shows the existence of the three isomers in the solution (two isomers with *D*_2*d*_ symmetry and one isomer with *C*_2*v*_ symmetry; see the Supporting Information for details). A DOSY experiment confirmed all the isomers had identical diffusion coefficients of 3.016×10^−10^ m^2^ s^−1^ (Figure S21). Additional ^1^H NMR signals that show exchange with the signals of the main *D*_2*d*_ isomer are observed at 238 K and are found to originate from a (much faster) second dynamic process, namely torsional motion of the ethylene bridges that slows down to about 1 s^−1^ at 238 K.

The ^31^P NMR spectrum of **3** in CD_3_CN at 238 K shows a slightly broadened major signal at *δ*=+1477.9 ppm and two minor signals at *δ*=+1492 and +1470 ppm (Figure S10), again reflecting the presence of conformational isomers; a similar pattern of signals could also be seen in [D_7_]DMF (Figure S14). With increasing temperature, the signal at lowest field broadens in both solvents and reaches coalescence with the main signal. A ^31^P EXSY spectrum (Figure S19) shows exchange between the two signals (about 300 s^−1^ at 273 K), with the population and exchange rates matching those of the isomers that feature different torsion of the ethylene bridge, detected in the ^1^H NMR spectra at 238 K. With a remarkable chemical shift of around *δ*=+1480 ppm, the P nucleus in **3** is even more deshielded than in the {Fe_2_P_2_} complex **A** (*δ*=+1407 ppm).6b

Monitoring of the reaction of **1** and Na(OCP) in [D_7_]DMF (where **1** and both products **2** and **3** are soluble) by ^31^P NMR spectroscopy at room temperature already showed only the signals for **2** and **3** (in the expected 1:1 product ratio) in the first spectrum few minutes after mixing at 238 K (Figure S26). This suggests that the reaction is too fast to detect any intermediates by this method. One may speculate that an initial product [LFe(PCO)(NCMe)]^+^ is formed that rapidly releases CO, and the resulting [LFeP(NCMe)]^+^ then reacts with starting complex **1** to give **3** (CO release from putative [LFe(PCO)(NCMe)]^+^ may also be triggered by association with **1**, without any terminal phosphido intermediate); the substitution of labile MeCN in another [LFe(PCO)(NCMe)]^+^ by the released CO appears to stabilize the *trans*‐PCO^−^ ligand in **2**. A ^31^P NMR spectrum recorded after completion of the reaction confirmed that **2** and **3** are formed in equal amounts (Figure S28).

Monitoring the reaction of **1** and Na(OCP) in [D_7_]DMF by UV/Vis spectroscopy showed the disappearance of the band at *λ*_max_=444 nm that is characteristic for **1** and the appearance of two intense low‐energy absorptions at 797 nm (*ϵ*=1.625×10^4^ L mol^−1^ cm^−1^) and 648 nm (*ϵ*=0.767×10^4^ L mol^−1^ cm^−1^; Figure S38). TD‐DFT calculations allowed these prominent bands to be assigned to electronic transitions within the Fe‐P‐Fe core of **3** (see below).

The redox properties of **3** in MeCN solution were studied by cyclic voltammetry (CV) and square‐wave voltammetry (SWV), which revealed two reversible reduction processes at *E*_1/2_=−1.95 and −2.26 V (versus Fc/Fc^+^) as well as an oxidation at *E*_1/2_=−0.6 V that appears reversible at rt and common scan rates (Figure 4). The first reduction and the oxidation of **3** are significantly shifted to lower potentials compared to the corresponding redox processes of the μ‐oxodiferric complex [(LFe)_2_(μ‐O)](OTf)_4_, which shows reduction waves at −1.33 and −2.03 V and a first oxidation at +0.87 V. UV/Vis spectroelectrochemical monitoring of the oxidation of complex **3** at an applied potential of −0.1 V (versus Fc/Fc^+^) showed the disappearance of the signatures of **3** at *λ*=797 and 648 nm and the concomitant appearance of a new band at *λ*=411 nm with two isosbestic points at *λ*=393 and 582 nm. However, prolonged electrolysis at the same potential (−0.1 V) revealed further changes reflected by new isosbestic points at *λ*=405 and 566 nm and a shift of *λ*_max_ from 411 to 423 nm (Figure 5). Subsequent reduction at −1.0 V confirmed the chemical reversibility of the two processes and regenerated the original spectrum of **3** (Figure S52). This result suggested that **3** can be oxidized twice to sequentially give new complexes **4** (*λ*_max_=411 nm) and **5** (*λ*_max_=423 nm). Bulk electrolysis of complex **3** at −0.1 V (versus Fc/Fc^+^, Figure S53) indeed indicated that two electrons are transferred at that potential (1.01 mmol, charge passed 0.196 C). To obtain further insight, CV and SWV measurements on **3** as well as **4** and **5** (which could both be isolated; see below) were performed at rt and −35 °C, and at variable CV scan rates (Figures S54–S56). Criteria such as the increasing separation of the current peaks for the forward and reverse scans (*i*_pf_, *i*_pr_) at lower temperatures (Figure S54, S55), the decrease of the function *i*_pf_/*v* ^1/2^ with increasing scan rate *ν* (Figure 4), as well as the appearance of a two‐humped peak in the SWV trace of the doubly oxidized **5** recorded at −35 °C suggest that two electrochemical processes are occurring around −0.6 V and are associated with chemical reactions, with the second oxidation of **3** likely being kinetically hindered.

**Figure 4:**
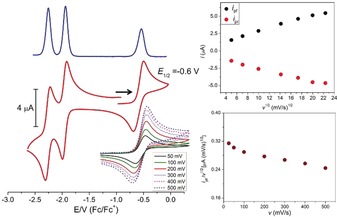
Left: CV (red) and SWV (blue) of **3** in MeCN/0.1 m [^*n*^Bu_4_N]PF_6_ at a scan rate of 200 mV s^−1^; the inset shows the CV of the process at −0.6 V at different scan rates. Right: plots of *i*_pf_ and *i*_pr_ versus *ν* ^1/2^ (top) and *i*_pf_/*ν* ^1/2^ versus the scan rate *ν* (bottom) for the process around −0.6 V. Fc/Fc^+^=ferrocene/ferrocenium couple.

**Figure 5:**
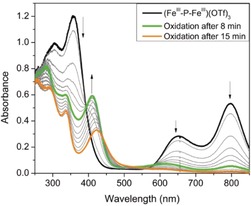
UV/Vis spectroelectrochemistry monitoring of the first and second oxidation of **3** in MeCN/0.1 m [^*n*^Bu_4_N]PF_6_ at an applied potential of −0.1 V (versus Fc/Fc^+^).

UV/Vis monitoring during a chemical redox titration of a green solution of **3** in MeCN with AgOTf (*E* ^0^(Ag^+^/Ag)=0.04 V versus Fc/Fc^+^)^20^ showed the same spectral changes: the addition of 1 equiv AgOTf gives a pale green solution of **4** (*λ*_max_=411 nm; *ϵ*=1.35×10^4^ mol L^−1^ cm^−1^) and addition of a further 1.5 equiv AgOTf leads to further oxidation to give **5** (*λ*_max_=423 nm; *ϵ*=1.05×10^4^ mol L^−1^ cm^−1^). However, whereas the first step is fast (within 5 min at rt and 15 min at −35 °C), the second step is kinetically hindered and is completed only after 12 h at −35 °C; the final product **5** appears to be unstable and gradually degrades at −10 °C to yield an unknown species with *λ*_max_=341 nm (Figure S42).

Bulk oxidation of **3** in MeCN with 1.0 or 2.5 equiv AgOTf and subsequent precipitation with Et_2_O allowed both the singly and doubly oxidized complexes [(LFe)_2_P(MeCN)](OTf)_4_ (**4**) and [(LFe)_2_P(MeCN)_2_](OTf)_5_ (**5**), respectively, to be isolated; in the latter case the temperature was kept at −35 °C throughout. Single‐crystalline material of both new compounds was obtained by the slow diffusion of Et_2_O into solutions of the crude material in MeCN. The molecular structures of the cations [(LFe)_2_P(MeCN)]^4+^ and [(LFe)_2_P(MeCN)_2_]^5+^ are included in Figure 3. Unfortunately, the quality of the crystallographic data were poor in the case of **4**; although the overall structure was clearly established, we refrain from any discussion of metric parameters. The core structure with an essentially linear Fe‐P‐Fe dumbbell is preserved throughout the series, but oxidation goes along with an increase in the coordination number of the iron ions because of the binding of MeCN ligands *trans* to the μ‐P bond. Hence, mixed‐valent **4** features a five‐ and a six‐coordinate metal ion, while both metal ions are six‐coordinate in **5**. This leads to a slight elongation of the Fe−P and Fe−C^NHC^ bonds in oxidized **5** compared to parent **3** (Table 1). It can be concluded that MeCN coordination/dissociation is the chemical process associated with the redox couples, which leads to electrochemical irreversibility in the cyclic voltammagrams recorded at low temperatures.

**Table 1:** Selected bond lengths and angles for complexes **3** and **5**.

	**3** [Å]/degree	**5** [Å]/degree
Fe1−C	1.953(8)–2.021(7)	1.994(3)–2.046(2)
Fe2−C	1.951(7)–2.008(7)	1.993(3)–2.040(3)
Fe1−P	1.993(2)	2.0079(7)
Fe2−P	1.998(2)	2.0016(7)
Fe1−N	–	1.995(2)
Fe2−N	–	2.013(2)
Fe1‐P‐Fe2	178.25(12)	179.61(4)

Mössbauer spectra of solid samples of **4** and **5** are included in Figure 3. As expected, the doubly oxidized species **5** shows a single quadrupole doublet (*δ*=0.00, Δ*E*_Q_=2.69 mm s^−1^) whereas nonsymmetric mixed‐valent **4** gives rise to two quadrupole doublets of equal intensity (*δ*=0.02, Δ*E*_Q_=1.96 mm s^−1^ and *δ*=0.01, Δ*E*_Q_=2.68 mm s^−1^). Comparison with the spectra of **3** and **5** allows the former doublet with a smaller quadrupole splitting to be assigned to the five‐coordinate iron and the latter doublet with a larger quadrupole splitting to the six‐coordinate iron center in **4**. Interestingly, the IS is essentially invariant for the entire redox series (Table 2). Although a cursory interpretation may suggest that the oxidation of **3** is purely ligand centered, it should be noted that the IS, which is predominantly a function of the 4s electron contribution to the electron density at the iron nucleus, is influenced by several factors, such as the 4s population, the shielding effect by the 3d population, and, more importantly, the Fe−ligand bond lengths that affect the radial extension of the 4s wave function.^21^ Hence the commonly observed negative correlation between the IS and oxidation state of the iron center (namely, IS is more negative for higher oxidation states) is not strictly valid and may even be reversed in certain cases.^21^ It has been established for a series of {FeNO}^*x*^ complexes based on tetracarbene macrocycle L that the Mössbauer‐derived IS cannot be used for a reliable assignment of the oxidation states.13d Specifically, the longer Fe−C^NHC^ and Fe−P bonds in oxidized **5** lead to reduced σ interactions and, therefore, a less compressed 4s orbital and lower 4s electron density at the iron nucleus. This may counterbalance the effect resulting from depopulating the d orbitals (and decreasing the 3s/4s shielding) upon oxidation.

**Table 2:** Experimental and DFT calculated (in brackets) ^57^Fe Mössbauer parameters for **3**, **4**, and **5**.

	**3**	**4**	**5**
*δ* [mm s^−1^]	0.01 (0.10/0.11)	0.02/0.01 (0.09/−0.04)	0.00 (−0.07/−0.07)
Δ*E*_Q_ [mm s^−1^]	1.94 (1.74/1.68)	1.96/2.68 (1.72/2.12)	2.69 (2.53/2.52)

SQUID magnetometry on solid samples of **4** and **5** revealed that singly oxidized **4** is an *S*=1/2 system (χ_M_ *T*=0.31 cm^3^ mol^−1^ K or *μ*_eff_=1.57 μ_B_ at 295 K; Figure S49), whereas doubly oxidized **5** has a well‐separated *S*=0 ground state and is diamagnetic over the entire temperature range (2–250 K; Figure S50), just like the parent complex **3**. The EPR spectrum of a frozen solution of **4** in MeCN at 163 K (Figure 6) shows an axial spectrum with *g*_*x,y*_=2.1059 and *g_z_*=2.0022 (i.e. *g*_z_≈2 and *g*_x,*y*_>2) and pronounced hyperfine coupling with the ^31^P nucleus [*A*(^31^P)=268.48 MHz, 271.61 MHz]. This indicates that the singly occupied molecular orbital (SOMO) contains some P contribution.

**Figure 6:**
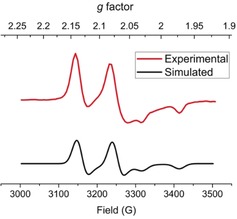
Experimental (top) and simulated (bottom) EPR spectra of **4** in frozen MeCN (163 K).

The ^1^H NMR spectrum of diamagnetic **5** in CD_3_CN at 238 K shows only a single set of resonances, which indicates that only a single isomer exists or that the interconversion of isomers is fast and more facile than in **3** (Figures S30–S35). The ^31^P NMR spectrum of **5** shows a slightly broadened signal at *δ*=+1122 ppm, which is shifted to a lower frequency than that of **3** (Figure S36). The ^1^H NMR spectrum of **4** shows that the compound is paramagnetic, in line with the SQUID and EPR data (Figure S49, S51).

To rationalize the observed spectroscopic signatures and trends, the electronic structures of complexes **3**–**5** were examined computationally (see the Supporting Information for details). The DFT results successfully reproduced key geometric parameters and spectroscopic properties within the uncertainty of the computations (Tables S2 and S3), thus lending credence to the proposed electronic structures discussed below.

As shown in Figure 7, the bonding of the (Fe‐P‐Fe)^3+^ core in **3** entails two π bonds involving the Fe‐d_*xz*_, Fe‐d_*yz*_, P‐p_*x*_, and P‐p_*y*_ orbitals, and one σ‐bond formed by the Fe‐$d_{z^{2}}$ and P‐p_*z*_ orbitals, similar to that found for linear triatomic molecules such as CO_2_. As the system possesses an effective inversion center, only P‐p_*z*_ can interact with the antisymmetric combination of the Fe‐$d_{z^{2}}$ orbitals. The same holds true for the P‐p_*x*_ and P‐p_*y*_ orbitals. As such, the symmetric combinations of the Fe‐$d_{z^{2}}$ , Fe‐d_*xz*_, and Fe‐d_*yz*_ orbitals are essentially nonbonding in nature, but the one constructed from the two Fe‐$d_{z^{2}}$ orbitals is situated slightly higher in energy because of the very weak interaction with the P‐s atomic orbital. In the upper valence region of **3**, in addition to the two Fe‐d_*xy*_ orbitals, all the Fe‐P‐Fe bonding and nonbonding molecular orbitals (MOs) are doubly occupied. Notably, the interaction within the Fe‐P‐Fe core has substantial covalent‐bond character, as evidenced by the contributions of the P atom in the σ‐ and π‐bonding MOs being 38 % and 50 %, respectively. In this situation, it is difficult to unambiguously assign physical oxidation states to the metal ions, although complex **3** can be formally interpreted as having two antiferromagnetically coupled low‐spin ferric centers bridged by a P^3−^ ion. The highly covalent Fe‐P‐Fe interaction accounts for the exceedingly strong antiferromagnetic coupling determined experimentally.

**Figure 7:**
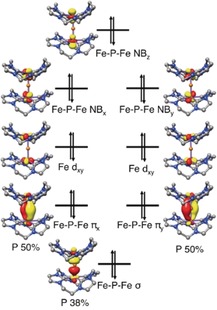
Molecular orbital diagram of complex **3**. NB=nonbonding.

As a consequence of the lack of inversion symmetry in complex **4**, the original Fe‐P‐Fe nonbonding MOs in **3** acquire some P‐p character and polarize toward Fe2 in **4** (Figure 8). Despite this, comparison of the electronic structures of complexes **3**–**5** (Figures 7, 8, and S63) reveals that upon oxidation, the two electrons that reside in the nonbonding MO of **3** with the dominant Fe‐$d_{z^{2}}$ contribution are successively removed. Alternatively, one can rationalize this notion as follows: the energy of this MO should be considerably raised by the binding of one or two MeCN molecules to the open axial sites of the Fe ions, because MeCN is a moderate σ donor and a weak π acceptor. As a consequence, this MO likely functions as the electron‐donating orbital upon oxidation. Taken together, the conversion of **3** into **4** and further into **5** is best described as two sequential metal‐centered redox processes. The redox‐active orbital is a nonbonding MO with respect to the Fe‐P‐Fe interaction; therefore, the Fe‐P‐Fe core is expected to remain largely intact during oxidation. This is in line with the geometric structures determined experimentally and computationally (Tables 1 and S2). It also accounts for the nearly identical Mössbauer IS measured for complexes **3**–**5**, because, typically, the bond distance between Fe and the strong ligands is a more important factor than the d‐electron count for determining the IS.^21^, ^22^ The variation in the QS measured for **3**–**5** clearly reflects the variations in the coordination numbers of the Fe sites.

**Figure 8:**
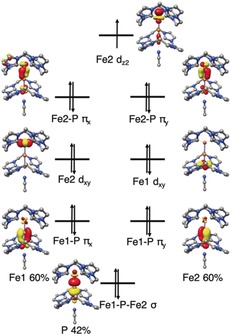
Molecular orbital diagram of complex **4**.

As shown in Figure 8, **4** features a Fe‐$d_{z^{2}}$ ‐centered SOMO that is situated slightly higher in energy than the two fully populated MOs, which possess substantial Fe‐d_*xz*_ and Fe‐d_*yz*_ character and are nearly degenerate. This bonding situation is consistent with the EPR spectrum of **4** with *g*_z_≈2 and *g*_x,*y*_>2, similar to a d^9^ ion in a trigonal coordination environment with the SOMO being the $d_{z^{2}}$ orbital.^23^ The SOMO contains marginal contributions from the P‐s and P‐p_*z*_ orbitals, in accord with the ^31^P hyperfine coupling detected by EPR spectroscopy.

TD‐DFT calculations suggest that the characteristic low‐energy absorption of **3** at *λ*=797 nm (Figure S61, Table S3) arises from transitions from the nonbonding Fe‐$d_{z^{2}}$ combination to its σ‐antibonding combination, while the band at *λ*=648 nm stems from promotion of an α electron from the nonbonding combinations of Fe‐d_*xz*_ and Fe‐d_*yz*_ to the same electron‐accepting σ* orbital (see Figure S62). The higher intensity of the former absorption originates from more favorable “overlap” between the electron‐donating and ‐accepting orbitals.

In summary, a first example of a Fe‐(μ‐P)‐Fe complex with a “naked” bridging phosphido ligand has been isolated, which complements the prominent ferric systems with Fe‐(μ‐O)‐Fe and Fe‐(μ‐N)‐Fe motifs. The macrocyclic tetracarbene scaffold supports the Fe‐(μ‐P)‐Fe core in at least three oxidation states, formally described as ranging from Fe^III^Fe^III^ to Fe^IV^Fe^IV^ (reduced congeners of **3** are still being studied). As a result of the very covalent Fe‐P‐Fe interaction, however, the physical oxidation state of the metal ions cannot be unambiguously assigned, but the oxidation is clearly metal‐based. Complexes with even numbers of electrons are effectively diamagnetic and the mixed‐valent Fe^III^Fe^IV^ species has a *S*=1/2 ground state. Sequential twofold oxidation of **3** removes electrons from a nonbonding orbital that originates from the symmetric Fe‐$d_{z^{2}}$ combination, which leaves the structure of the Fe‐(μ‐P)‐Fe core essentially unperturbed. This study underlines the usefulness of the macrocyclic tetracarbene ligand scaffold for isolating unusual Fe (and other 3d metal) complexes, and the potential of Na(OCP) to serve as a P‐anion transfer reagent. Future work will address the reductive chemistry of **3**, and will focus on elucidating the reactivity patterns of the Fe‐(μ‐P)‐Fe motif in its various oxidation states.

## Conflict of interest

The authors declare no conflict of interest.

## Supporting information

As a service to our authors and readers, this journal provides supporting information supplied by the authors. Such materials are peer reviewed and may be re‐organized for online delivery, but are not copy‐edited or typeset. Technical support issues arising from supporting information (other than missing files) should be addressed to the authors.

Supplementary
