# Supplementary material for: A μ‐Phosphido Diiron Dumbbell in Multiple Oxidation States
Source: Angew Chem Int Ed Engl. 2019 Aug 23;58(40):14349–56. doi: 10.1002/anie.201908213 (PMC6790664; doi:10.1002/anie.201908213)
Supplement: Supplementary file 1 — Supplementary [file ANIE-58-14349-s001.pdf]

Angewandte Chemie
Eine Zeitschrift der Gesellschaft Deutscher Chemiker

Supporting Information

A μ-Phosphido Diiron Dumbbell in Multiple Oxidation States

Munmun Ghosh, Hanna H. Cramer, Sebastian Dechert, Serhiy Demeshko, Michael John, Max M. Hansmann, Shengfa Ye, and Franc Meyer**

anie_201908213_sm_miscellaneous_information.pdf

Table of Contents

1. General Considerations and Equipment Used....S2
2. Synthetic Protocols for Complexes....S3
3. NMR Spectra for 2....S5
4. NMR Spectra for 3....S10
5. NMR Spectra for 4....S22
6. NMR Spectra for 5.... S23
7. IR Spectra....S28
8. UV-vis Spectra....S29
9. ESI Mass Spectrometry....S33
10. Magnetic Susceptibility Measurements....S34
11. EPR Spectra....S36
12. (Spectro)Electrochemical Measurements....S37
13. X-ray Crystallography....S42
14. DFT Calculations....S47
15. References....S51

1. General Considerations and Equipment Used

All air-sensitive experiments were carried out under a dry nitrogen atmosphere using either a MBraun LabMaster glovebox or standard Schlenk techniques. Solvents were dried and degassed according to standard procedures before use. Deuterated solvents were dried over activated molecular sieves (4 Å for DMF-d_7, 3 Å for all other solvents) and degassed. $[LFe(NCMe)_2](OTf)_2$ (**1**)[1] and NaOCP[2] were synthesized according to literature procedures. All other reagents were purchased from commercial sources and used without further purification unless mentioned otherwise.

NMR spectra were recorded on a Bruker Avance III HD 500, a Bruker Avance III HD 400 and a Bruker Avance III 300. If not stated otherwise, all spectra were measured at 298 K. ^{1}H NMR and ^{13}C{^{1}H} NMR chemical shifts are reported in parts per million in relation to tetramethylsilane. ^{1}H NMR chemical shifts were referenced to the residual hydrogen atom signals of the used deuterated solvents and ^{13}C NMR chemical shifts were calibrated with the solvent's natural abundant ^{13}C resonances.[3]

UV-vis spectra were recorded on a Varian Cary 5000 instrument and a Varian Cary 8454.

IR spectra were measured with a Jasco FT/IR-4100 PikeGladi ATR spectrometer or with an Agilent Technologies Cary 630 FTIR spectrometer with Dial Path Technology and analyzed with FTIR MicroLab software.

Elemental analyses were carried out using an Elementar Vario EL III instrument by the analytical laboratory of the Institute of Inorganic Chemistry at the Georg-August-University Göttingen.

ESI mass spectrometry was performed on a Bruker HCT Ultra connected to an argon glovebox, or on a Bruker maXis ESI-QTOF.

Cyclic voltammograms were recorded inside a N_2-filled glovebox with a Gamry Reference 600 potentiostat using a gas-tight CV cell with glassy carbon as working electrode, a platinum wire as counter electrode and a silver wire as pseudo reference electrode. Cyclic voltammograms were referenced to the internal standard Fc/Fc^+ (Fc = ferrocene) that was added at the end of the experiment. All electrochemical measurements were performed in dry and degassed MeCN/0.1 M $[N^nBu_4]PF_6$ solutions at room temperature if not stated otherwise.

X-band EPR spectra were measured on a Bruker E500 ELEXSYS spectrometer equipped with a standard cavity (ER4102ST, 9.45 GHz) and simulated using the program EasySpin.[4]

Mößbauer spectra were recorded with a ^{57}Co source in a Rh matrix using an alternating constant acceleration Wissel Mößbauer spectrometer operated in the transmission mode and equipped with

a Janis closed-cycle helium cryostat. Isomer shifts are given relative to iron metal at ambient temperature. Simulation of the experimental data was performed with the Mfit program using Lorentzian line doublets.[5]

Magnetic susceptibility measurements were carried out with a *Quantum-Design* MPMS-XL-5 SQUID magnetometer equipped with a 5 Tesla magnet in the range from 295 or 250 to 2.0 K at a magnetic field of 0.5 T. The powdered samples were contained in a Teflon bucket and fixed in a non-magnetic sample holder. Each raw data file for the measured magnetic moment was corrected for the diamagnetic contribution of the Teflon bucket according to M^{dia}(bucket) = $\chi_g \cdot m \cdot H$, with an experimentally obtained gram susceptibility of the Teflon bucket. The molar susceptibility data were corrected for the diamagnetic contribution. Experimental data for were modelled by using a fitting procedure to the appropriate Heisenberg-Dirac-van-Vleck (HDvV) spin Hamiltonian for isotropic exchange coupling and Zeeman splitting:

$$\hat{H} = -2J\hat{S}_1\hat{S}_2 + g\mu_B\vec{B}\left(\vec{S}_1 + \vec{S}_2\right)$$

Temperature-independent paramagnetism (*TIP*) and a Curie-behaved paramagnetic impurity (*PI*) with spin S = 5/2 were included according to $\chi_{calc} = (1 - PI)\cdot\chi + PI\cdot\chi_{mono} + TIP$. Experimental data were modelled with the *julX* program.[6]

2. Synthetic Protocols for Complexes

Synthesis of [LFe(PCO)(CO)]OTf (**2**): [LFe(MeCN)$_2$](OTf)$_2$ (**1**) (40 mg, 51 µmol, 1 equivalent) was suspended in THF and kept at -35°C. A precooled THF solution of Na(OCP)(dioxane)$_{2.5}$ (8.5 mg, 30.6 µmol, 0.6 equivalent) was added dropwise. After few seconds a green precipitate started to appear, and the reaction mixture was kept at -35 °C for 12 hours and then stirred for another 12 hours at room temperature. The green precipitate was separated by filtration and the THF solution was layered with Et_2O (12 mL) to give yellow crystals of the product **2** (15 mg, 23.3 µmol, 45.9% with respect to **1**). **2** is soluble in CH_3CN and stable at room temperature. **^{1}H NMR** (500 MHz, CD_3CN, 298 K) δ [ppm]: 4.34-4.40 (m, 4H, CH_2CH_2), 4.82-4.89 (m, 4H, CH_2CH_2), 6.07 (d, J = 12.5, 2H, CH_2), 7.13 (dd, J = 12.5, 7.9, 2H, CH_2), 7.17 (d, J = 2, 2H, CH^{im}), 7.35 (d, J = 2, 2H, CH^{im}); **^{13}C NMR** (126 MHz, CD_3CN, 238 K) δ [ppm]: 50.63 (CH$_2$$CH_2$), 62.98 ($CH_2$), 123.06 ($C$H^{im}), 125.27 ($C$H^{im}), 168.88 ($J_{PC}$ = 94.4 Hz, PCO), 186.33 (d, J_{PC} = 4.5 Hz C^{im}), 222.22 (d, J_{PC} = 9.0 Hz CO); **^{31}P NMR** (202 MHz, CD_3CN, 298 K) δ [ppm]: -412.0; **UV-vis** λ_{max} [nm] (ε [L mol^{-1} cm^{-1}]): 327 (1.03×10^4); **ESI-MS** (positive ion mode, MeCN) *m*/*z* (%): 201.9 [LFe]$^{2+}$ (202.05), 490.9 [LFe(PCO)(CO)]$^+$ (491.08), 580.9 [LFe(CO)(OTf)]$^+$ (581.06).

Synthesis of [(LFe)$_2$P](OTf)$_3$ (**3**): The green precipitate separated from the above reaction was washed with THF (3 × 5 mL) and Et_2O (5 mL) and then dissolved in CH_3CN (3 mL). Slow Et_2O diffusion into the solution gave green crystals of **3** suitable for X-ray diffraction (20 mg, 34.2 µmol, 60% with respect to **1**). **^{1}H NMR** (500 MHz, CD_3CN, 238 K) δ [ppm]: 3.98 (dt, *J*=15.5, 5.0 Hz; 1H, H^a), 4.27 (dt, *J*=15.9, 3.3 Hz; 1H, $H^{a'}$), 4.47 (t, *J*=4.3 Hz, 2H, H^b), 5.66 (d, *J*=12.8 Hz, 1H, H^c), 5.80 (d, *J*=12.8 Hz, 1H, H^d), 7.08 (s, 1H, H^h), 7.09 (s, 1H, H^e), 7.13 (s, 1H, H^f), 7.24 (s, 1H, H^g). Other minor isomers (7% and 13% at 238 K) that differ by the mutual rotation of the two {LFe} units can be identified. **^{13}C NMR** (126 MHz, CD_3CN, 238 K) δ [ppm]: 48.56 (C^A), 52.76 (C^B), 62.46 (C^C), 121.21 (C^E), 121.70 (C^D), 122.63 (C^F), 124.59 (C^G), 180.94 (C^H), 191.45 (C^I). **^{31}P NMR** (202 MHz, CD_3CN, 238 K) δ [ppm]: +1477.9 (br); at 298 K signals for the two other rotational isomers are discernible at +1492 and +1470 ppm. NMR spectra have also been recorded in DMF-d7. **UV-vis** λ_{max} [nm] (ε [L mol^{-1} cm^{-1}]) 797 nm (1.625×10^4) and 648 nm (0.767×10^4). **ATR-IR**: 1761 (br); **ESI-MS** (positive ion mode, MeCN) *m/z* (%): 201.8 $[LFe]^{2+}$ (202.05), 279.5 $[LFePFeL]^{3+}$ (279.73), 434.9$[LFeP]^+$ (435.08), 552.9 $[LFe(OTf)]^+$ (553.06). Anal Calcd for $C_{39}H_{40}F_9Fe_2N_{16}O_9PS_3$: C, 36.41; H, 3.13; N, 17.42; Found C, 37.17; H, 3.38; N, 17.93.

Synthesis of [(LFe)$_2$P](OTf)$_4$ (**4**): To a solution of **3** (10 mg, 7.7 µmol) in MeCN (2 mL) was added AgOTf (2 mg, 7.7 µmol) at -35 ^{o}C, leading to the immediate formation of a precipitate. The reaction mixture was left stirring overnight and the solution colour changed to pale green. Any solid precipitate was removed by filtration through a glass filter. Slow diffusion of Et_2O into the resulting MeCN solution gave pale green coloured crystals after 5 days. **UV-vis** λ_{max} [nm] (ε [L mol^{-1} cm^{-1}]): 412 (1.35×10^4), 596 (1.55×10^3) (br). Anal Calcd for $C_{42}H_{43}F_{12}Fe_2N_{17}O_{12}PS_4$ (**4**·MeCN): C, 34.16; H, 2.93; N, 16.12; Found C, 34.70; H, 2.92; N, 16.42.

Synthesis of [(LFe)$_2$P](OTf)$_5$ (**5**): To a precooled solution of **3** (10 mg, 7.7 µmol) in MeCN (2 mL) was added AgOTf (5 mg, 2.5 equivalents, 19.25 µmol) at -35 ^{o}C. The solution colour changed to pale green and after 12 hours at -35 ^{o}C the solution turned brown. Any precipitate was separated by filtration through a glass filter while keeping the solution at at -35 ^{o}C throughout. Slow diffusion of Et_2O into the resulting brown solution at -35 ^{o}C gave brown coloured crystals suitable for X-ray diffraction. **^{1}H NMR** (500 MHz, CD_3CN, 238 K) δ [ppm]: 3.91 (dd, 4H -CH_2CH_2), 4.04 (dd, 4H -CH_2CH_2), 5.68 (d, 2H, -CH_2), 6.47 (d, 2H, -CH_2) 7.30 (d, 4H, -CH^{im}), 7.78 (d, 4H, -CH^{im}). **^{13}C NMR** (126 MHz, CD_3CN, 238 K) δ [ppm]: 48.80 (-CH$_2$$CH_2$), 62.44 (-$CH_2$), 126.54 ($C$H^{im}), 126.69 ($C$H^{im}), 162.6 ($C^{im}$); **^{31}P NMR** (202 MHz, CD_3CN, 298 K) δ [ppm]: +1122; **UV-vis** λ_{max} [nm] (ε [L mol^{-1} cm^{-1}]): 424 nm (1.05×10^4).

3. NMR Spectra for 2

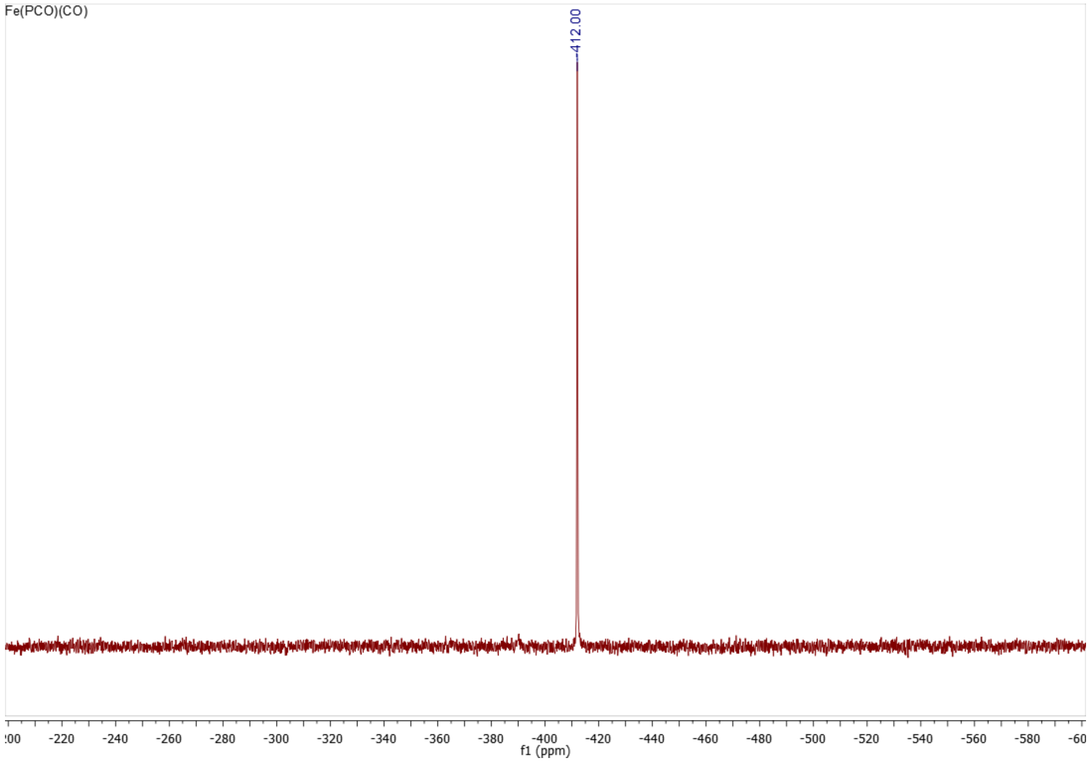

Figure S1: ^{31}P NMR spectrum of complex **2** in CD_3CN at room temperature

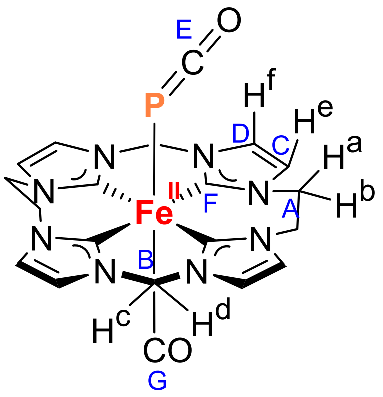

Numbering Scheme for **2** (for NMR assignment)

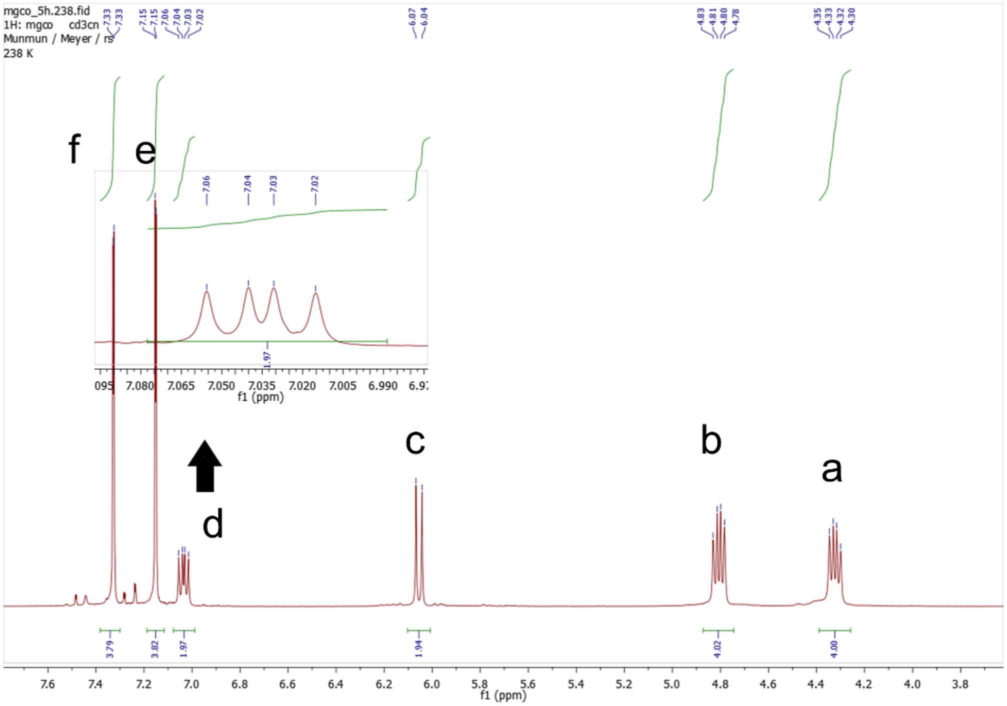

Figure S2: ^{1}H NMR spectrum of complex **2** in CD_3CN (the inset shows a magnification of the resonance of H^d to emphasize the ^{31}P coupling in comparison with the spectrum shown in Figure S3)

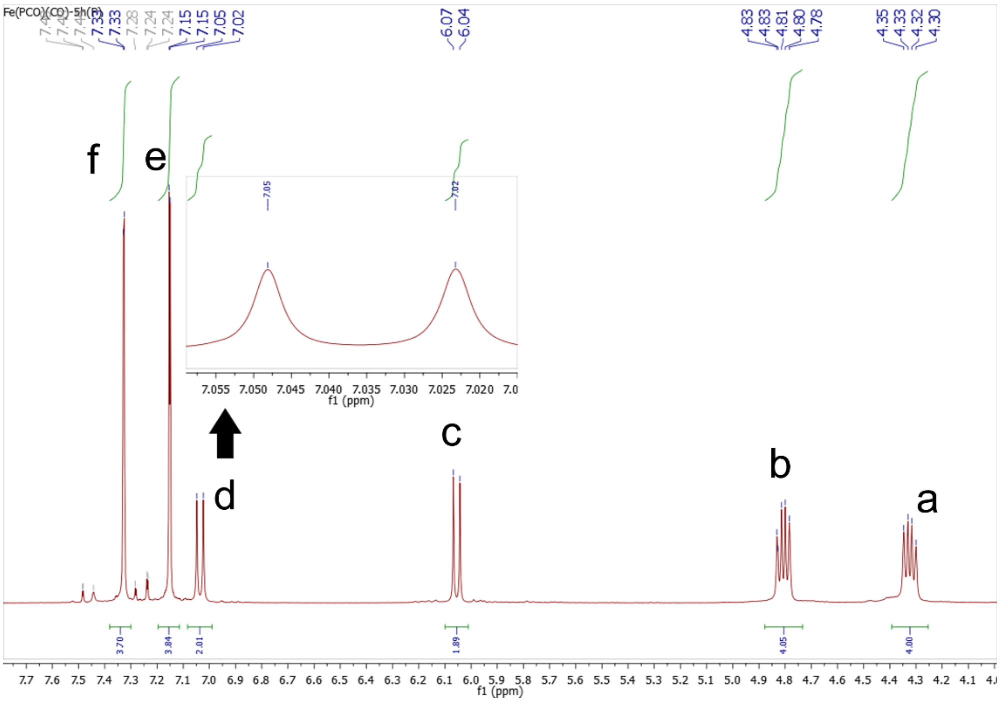

Figure S3: $^1H\{^{31}P\}$ NMR spectrum of complex **2** in CD_3CN

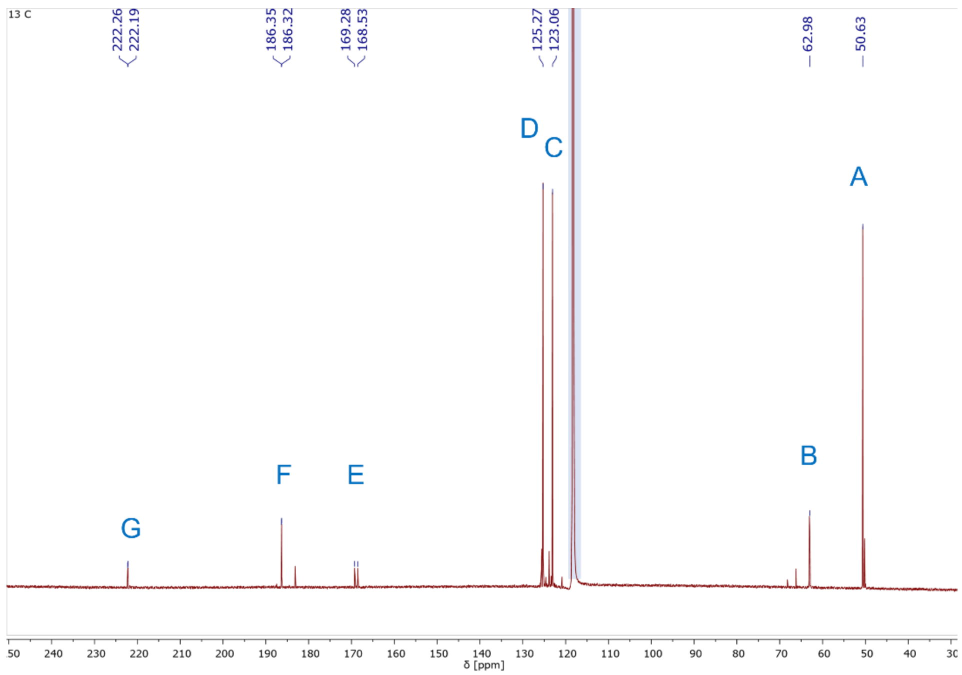

Figure S4: ^{13}C NMR spectrum of complex **2** in CD_3CN (the blue shaded signal corresponds to solvent peak).

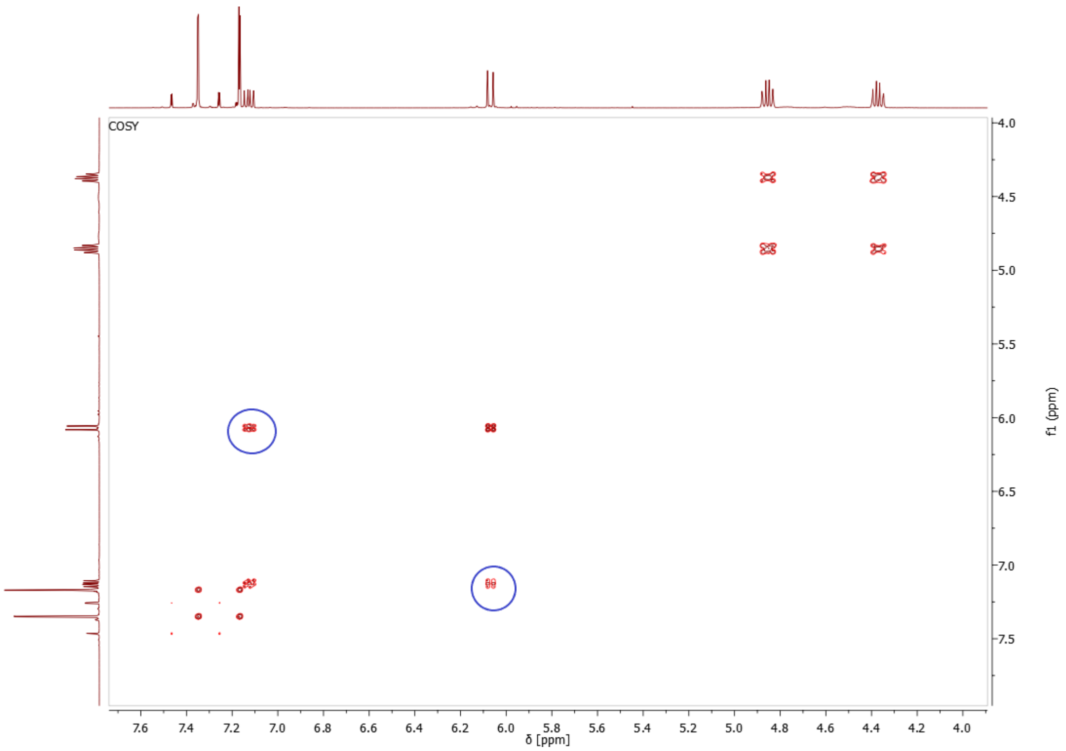

Figure S5: COSY NMR spectrum of complex **2** in CD_3CN (the blue circles are showing the correlations between –C*H*H and -CH*H* of the same carbon center)

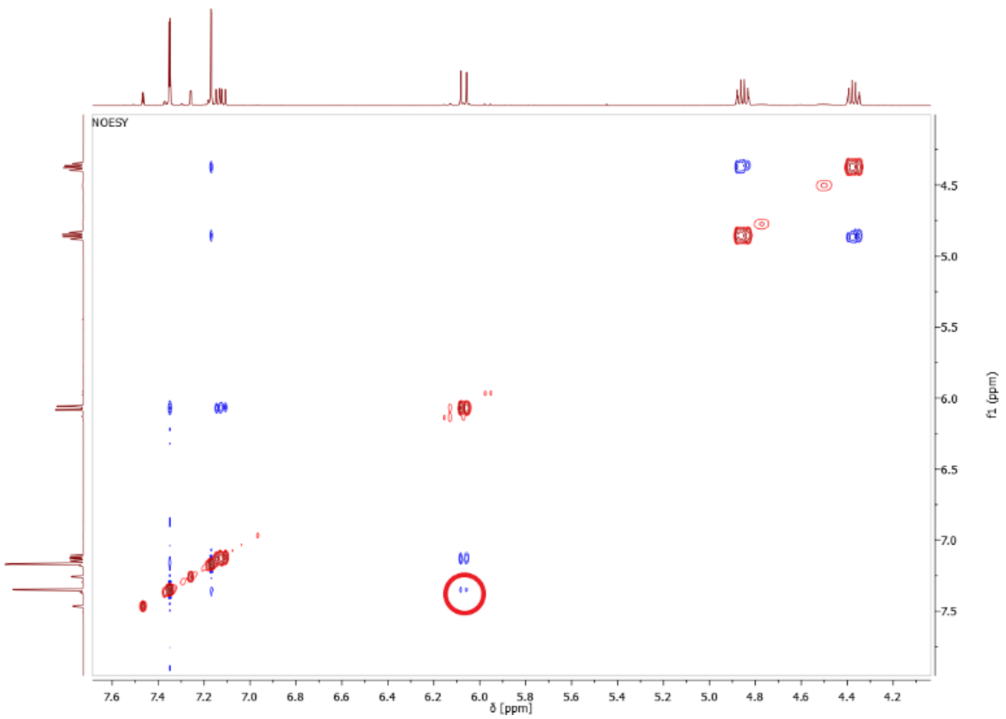

Figure S6: NOESY NMR spectrum of complex **2** in CD_3CN (the red circle is showing the correlation between the -CH_2 (at 6.07) and imidazole protons).

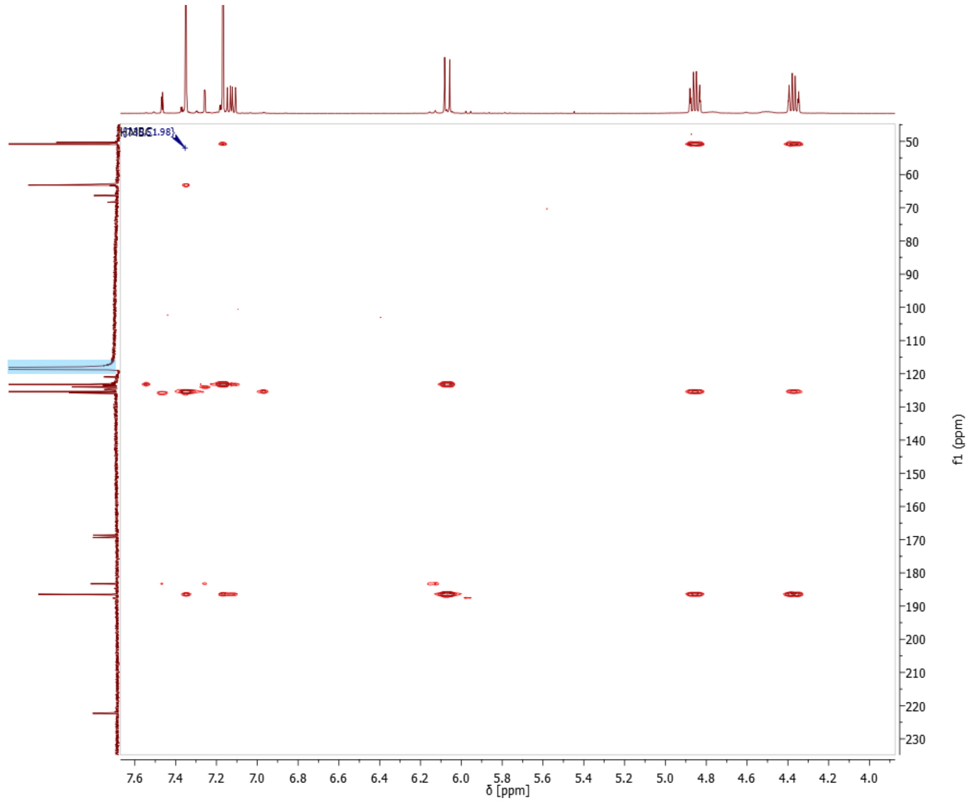

Figure S7: HMBC spectrum of complex **2** in CD_3CN (the blue shaded signal corresponds to the solvent peak).

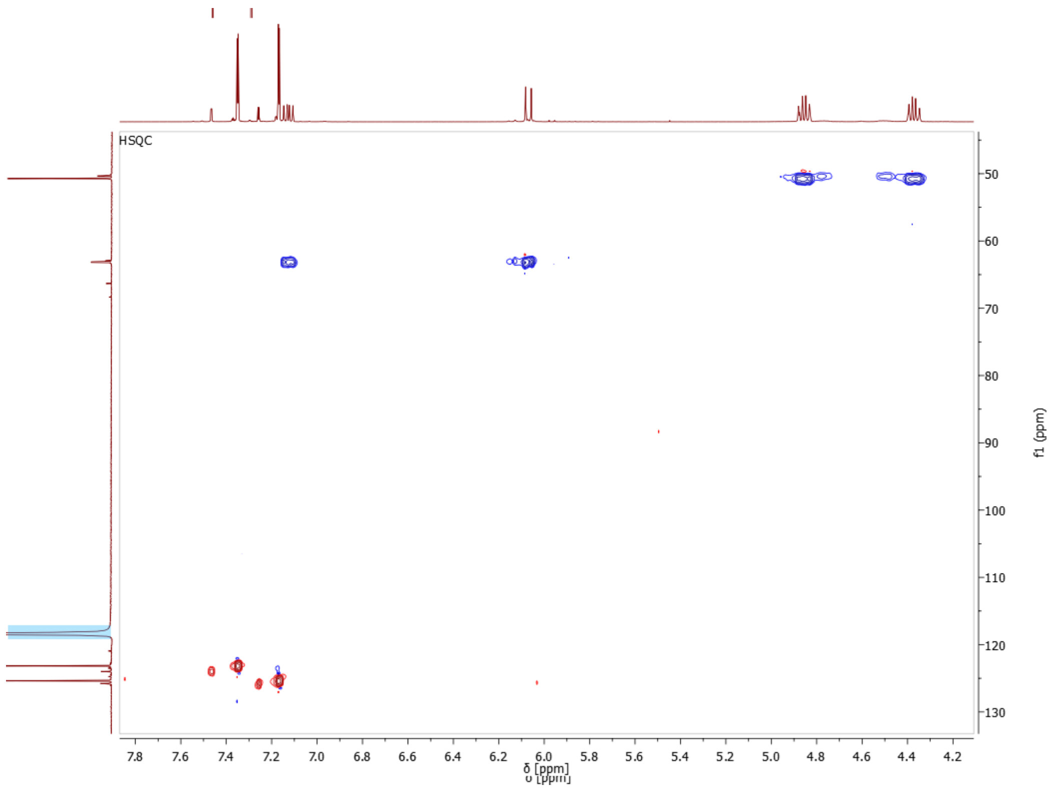

Figure S8: HSQC NMR spectrum of complex **2** in CD_3CN (the blue shaded signal corresponds to the solvent peak).

NMR Spectra for 3

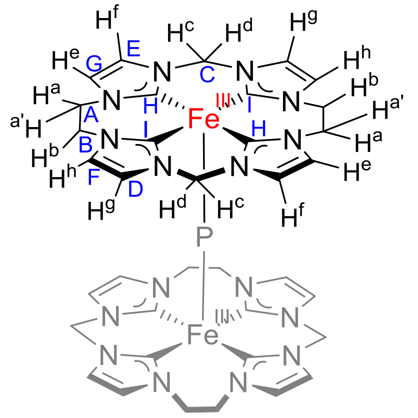

Numbering Scheme for **3** (for NMR assignment); the ligands L are symmetry related (D_{2d})

Three isomers are possible if one considers that the macrocyclic ligands adopt a saddle shaped conformation. Hence, there are two isomers possible in which the methylene bridges of one ligand are eclipsed with the ethylene bridge of the other ligand (effective D_{2d} symmetry at 298 K in solution): in one isomer the methylene bridges point inside towards the other ligand, and the ethylene bridges point outwards (like in the crystal structure), while in another isomer (in the manuscript termed 3rd isomer due to its low population of about 2%) it is vice versa, equivalent to both macrocycles being flipped. The third isomer (in the manuscript termed 2nd isomer, about 25% population) has the methylene groups eclipsed, equivalent to a 90° rotation and simultaneous flip of just one macrocycle with respect to the 1st isomer. Here, the two ligands become inequivalent (effective C_{2v} symmetry in solution) as the methylene groups of the unrotated ligand continue to point inwards, while those of the flipped ligand points outwards.

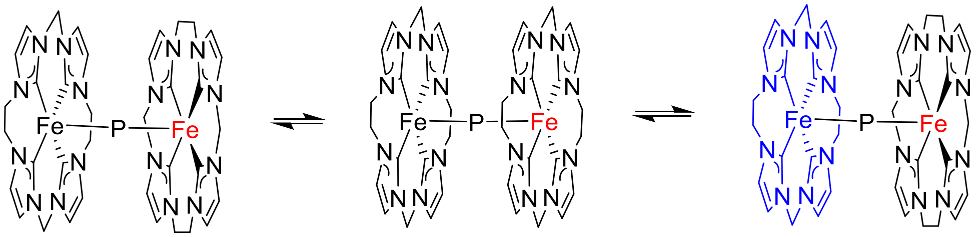

D_{2d} C_{2v} D_{2d} flipped

The successive macrocyclic ring flips can be observed in EXSY spectra of **3** at 298 K in both MeCN-d_3 and DMF-d_7 in the form of exchange peaks between axial and equatorial protons of different isomers, with rate constants between 0.1 and 0.5 s^{-1}. Hence, coalescence for this isomerization would be expected only above 100°C. Additional peaks observed at 238 K originate from a (much faster) second dynamic process, namely torsional motion within the ethylene bridges: This process slows down to about 1 s^{-1} at 238 K (exchange peaks between the two CH_2

units of the ethylene bridges) and reduces the symmetry from D_{2d} to D_2. The additional peaks exchange with the main peaks at the same rate and are assigned to a (overall C_2 symmetric) species with opposite N-C-C-N torsion angles of the two macrocycles, while the main D_2 symmetric species has four similar angles (as observed in the crystal structure).

Variable temperature ^{1}H and ^{31}P NMR spectra in different solvents (MeCN-d$_3$ and DMF-d$_7$) are shown in Figures S11 – S14. ^{1}H and ^{31}P EXSY spectra showing the expected cross peaks indicating exchange between all three isomers in both MeCN-d$_3$ and DMF-d$_7$ are shown in Figures S16 – S19.

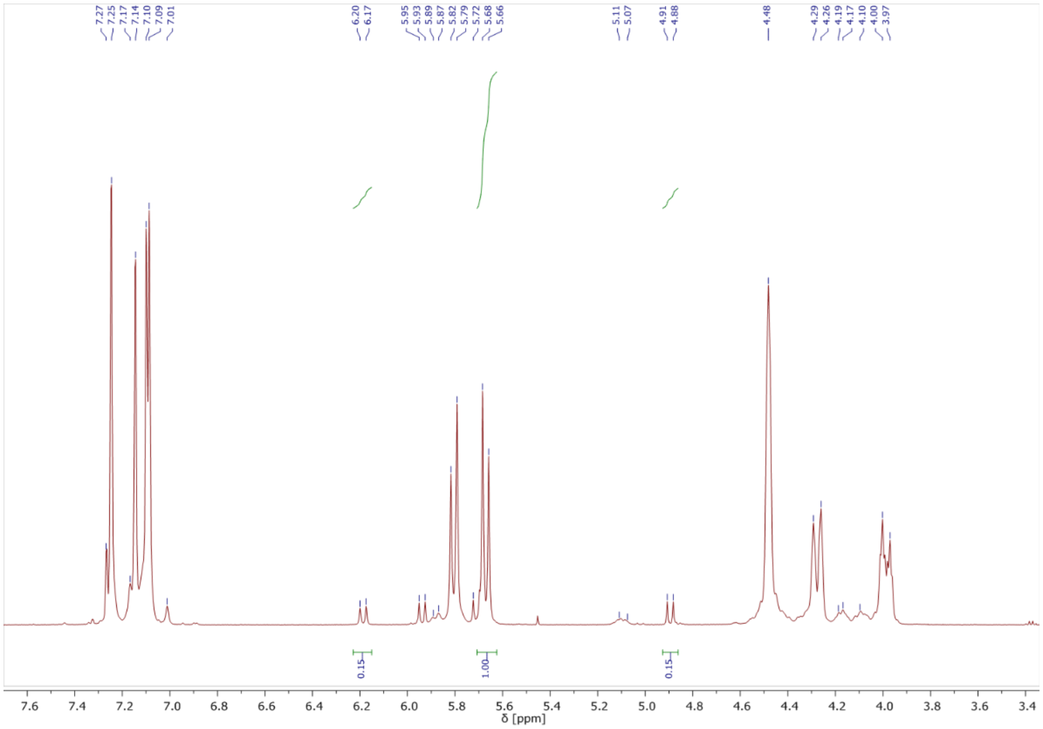

Figure S9: ^{1}H NMR spectrum of complex **3** in CD_3CN at 238 K

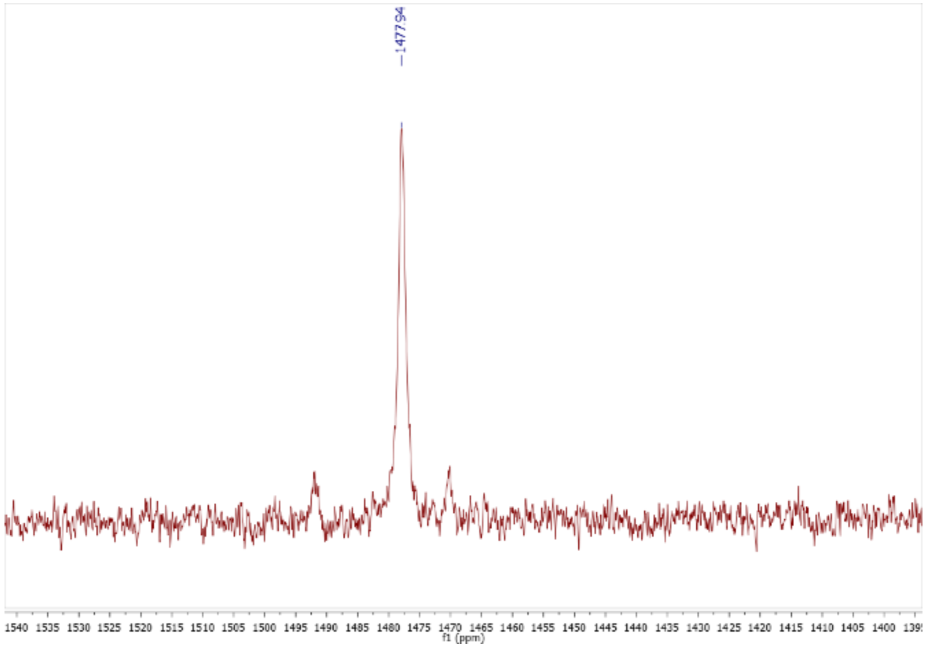

Figure S10: ${}^{31}P$ NMR spectrum of complex **3** in CD_3CN at 238 K; besides the main signal at +1477.9 ppm (br), two minor signals are detectable at +1492 and +1470 ppm. For variable temperature spectra in DMF-d_7 see Figures S13, S14.

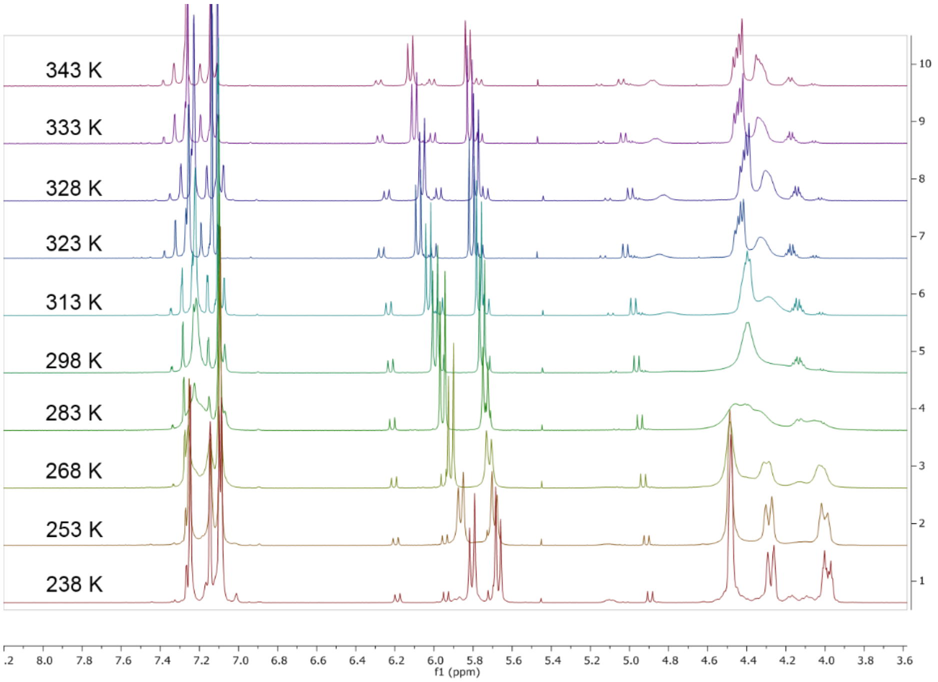

Figure S11: Variable temperature ${}^{1}H$ NMR spectra of complex **3** in the range from 238 K to 343 K in CD_3CN.

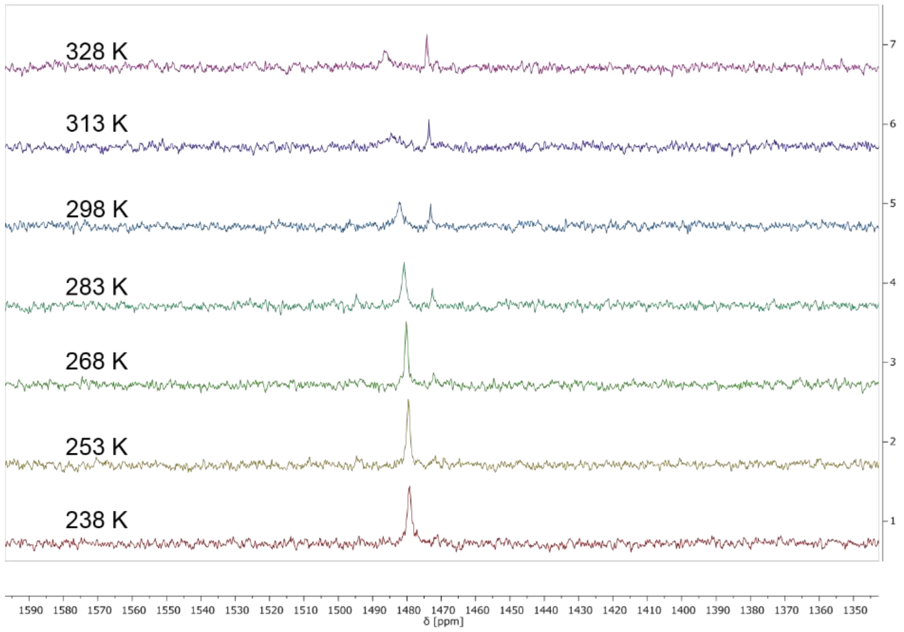

Figure S12: Variable temperature ^{31}P NMR spectra of complex **3** in the range from 238 K to 328 K in CD_3CN

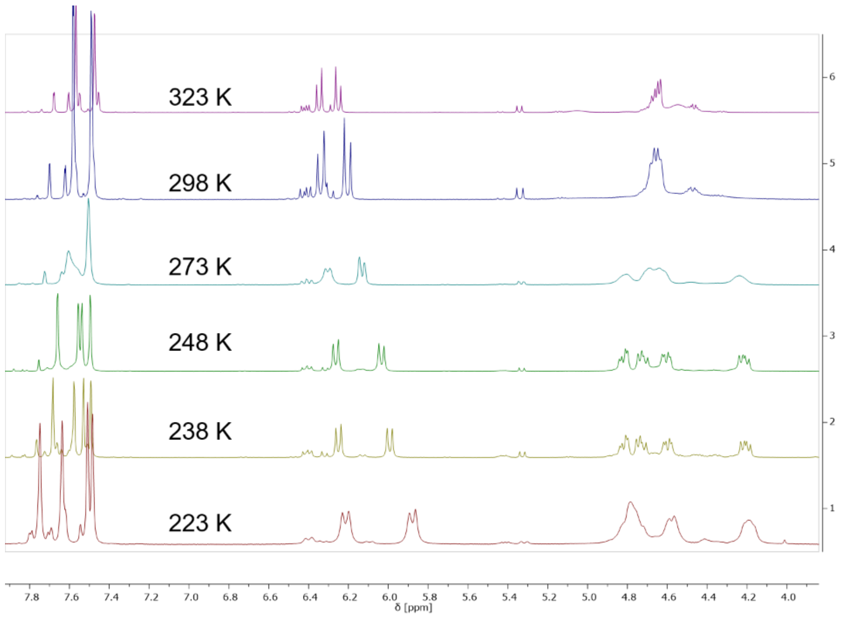

Figure S13: Variable temperature ^{1}H NMR spectra of complex **3** in the range from 223 K to 323 K in DMF-d_7.

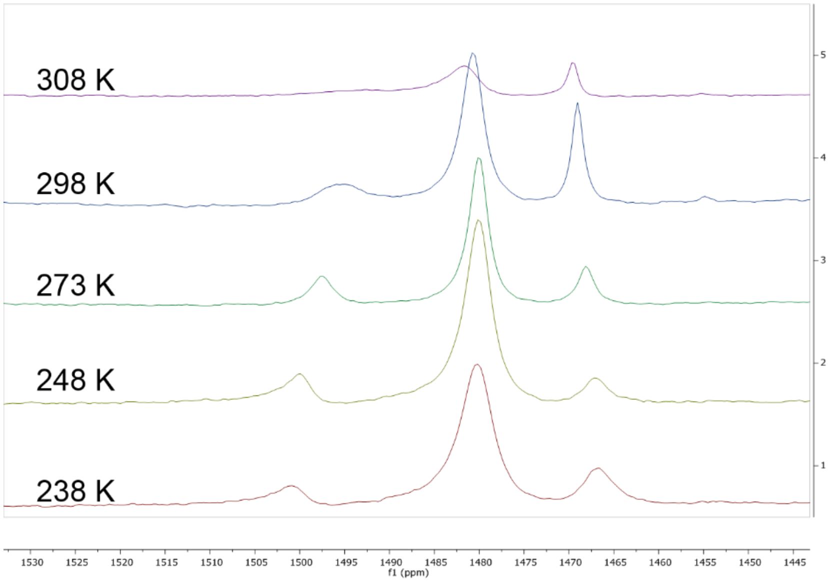

Figure S14: Variable temperature ^{31}P NMR spectra of complex **3** in the range from 238 K to 308 K in DMF-d7.

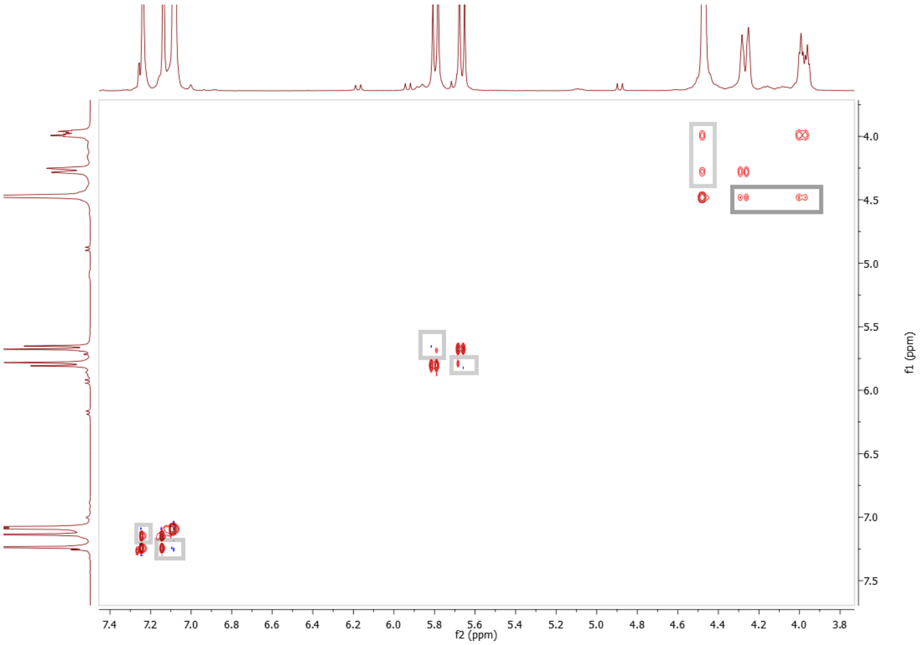

Figure S15: NOESY spectrum of complex **3** in CD_3CN at 238 K, showing the -CH_2CH_2 (4 – 4.5 ppm), -CH_2 (5.5 – 6 ppm) and imidazole-2-yliden (7 – 7.4 ppm) protons.

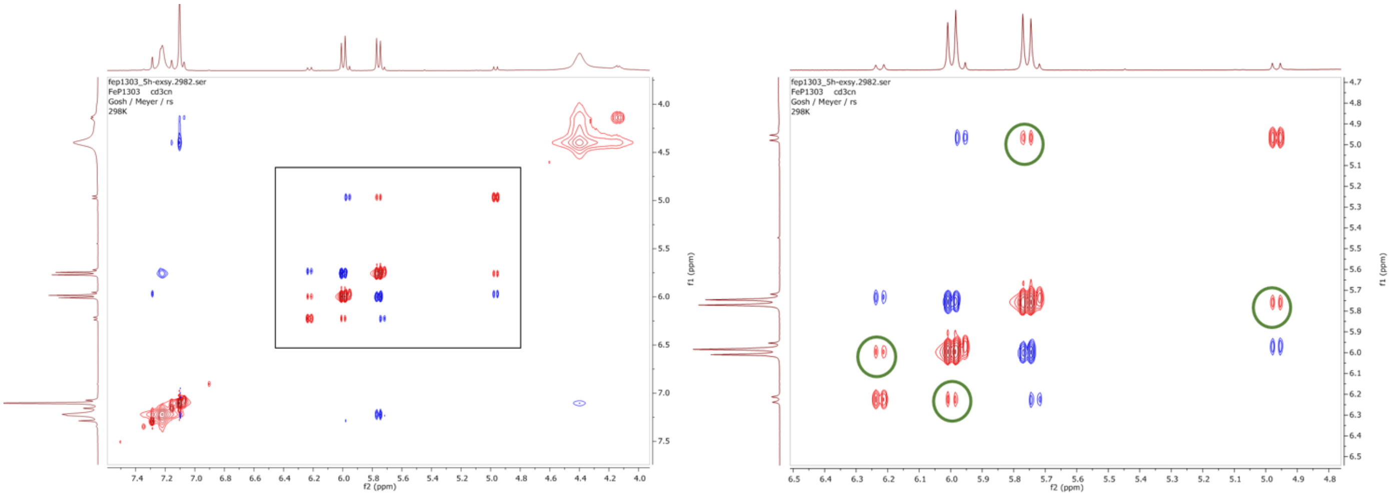

Figure S16: EXSY spectrum of complex **3** in CD_3CN at 298 K (left); a zoom of the grey shaded area is shown on the right side, reflecting the exchange between isomers.

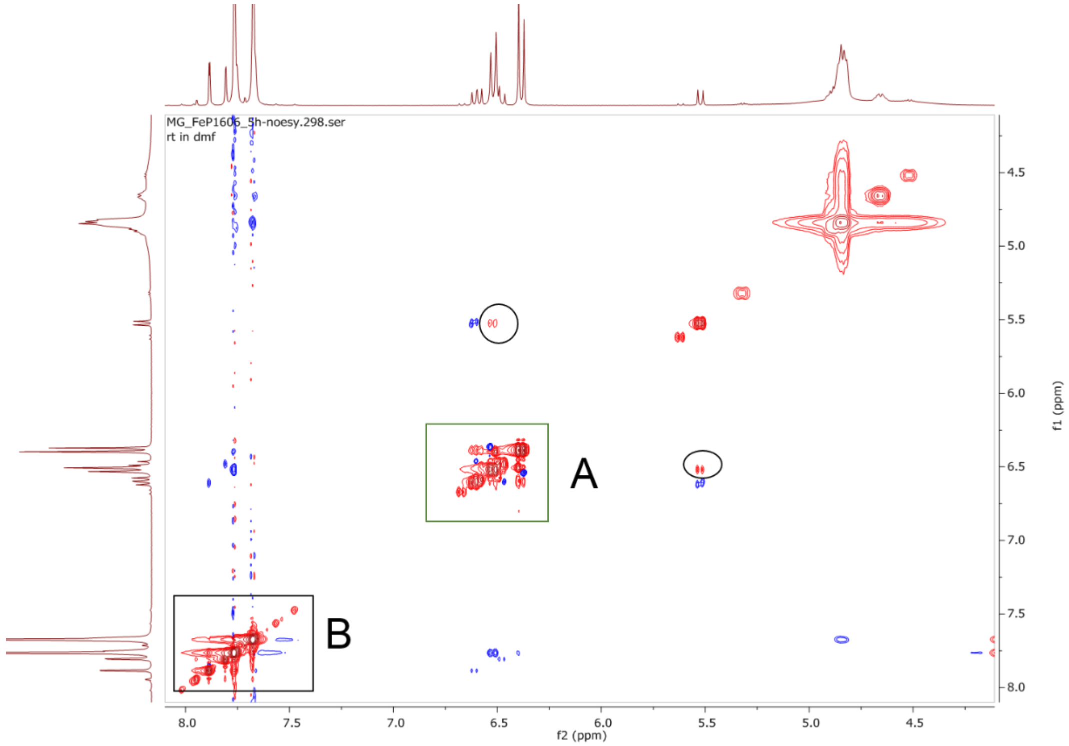

Figure S17: EXSY spectrum of complex **3** in DMF-d_7 at 298 K; zooms of parts A and B are shown in Figure S18.

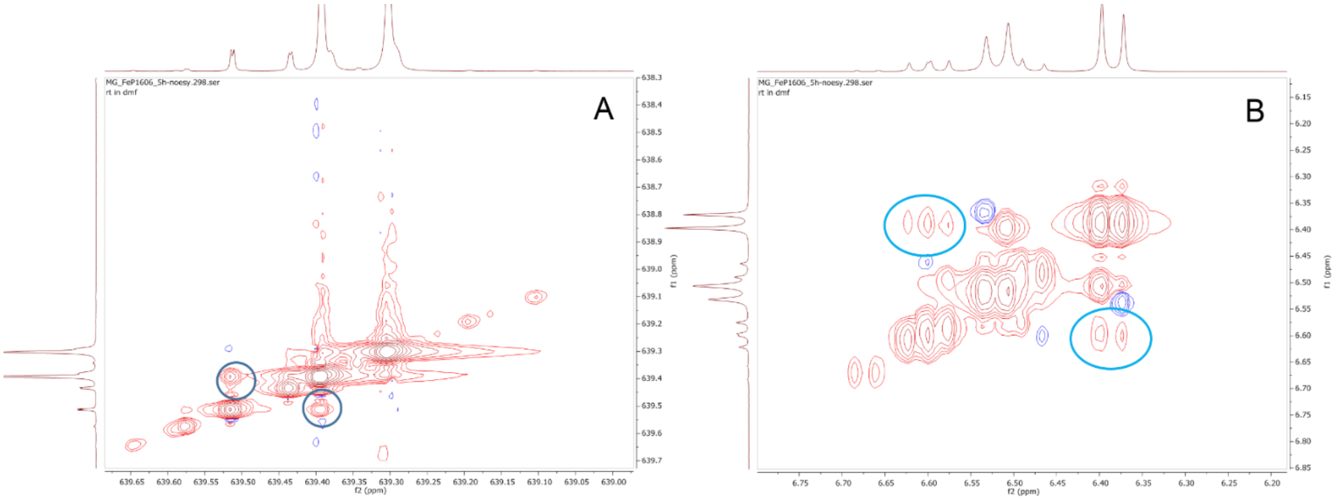

Figure S18: Zoomed parts A (left) and B (right) of the EXSY spectrum of complex **3** in DMF-d_7 at 298 K (compare Figures S17), reflecting the exchange of CH_2 protons (Figure A) and imidazole proton (Figure B) due to interconversion of isomers.

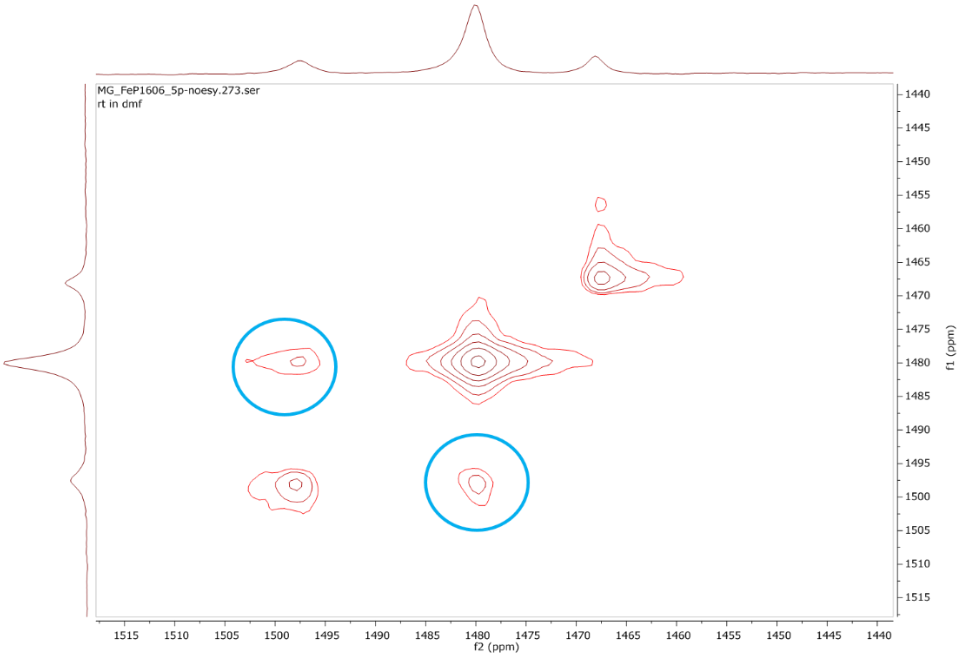

Figure S19: ^{31}P EXSY spectrum of complex **3** in DMF-d_7 at 273 K.

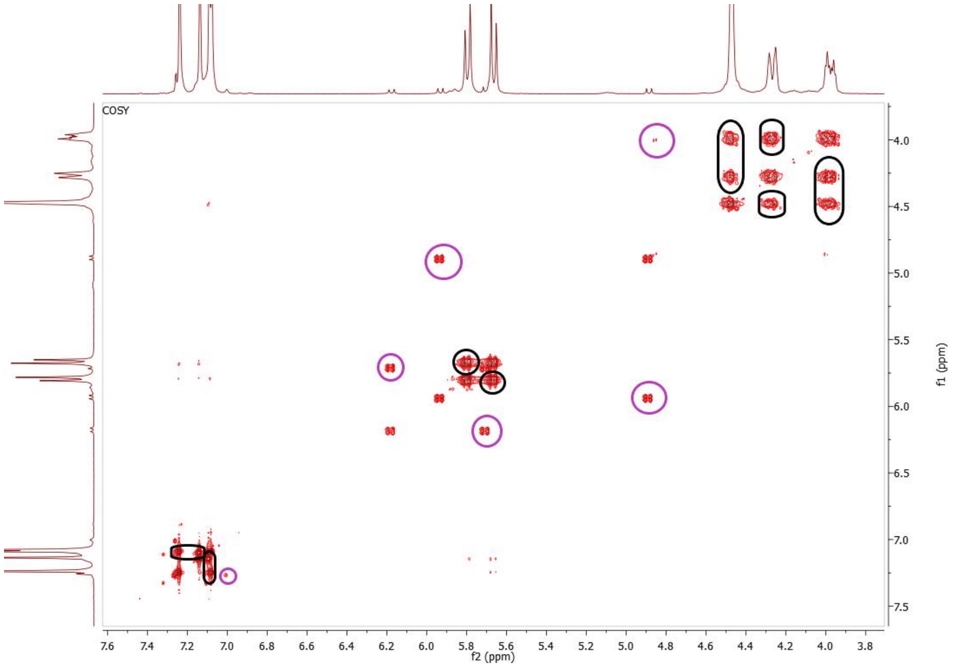

Figure S20: COSY NMR spectrum of complex **3** in CD_3CN at 238 K, showing correlations for the -*CH_2CH_2* (4 – 4.5 ppm), -*CH_2* (5.5 – 6 ppm) and imidazole-2-ylidene (7 – 7.4 ppm) protons for one isomer in black and for another isomer in violet circles (the third isomer is difficult to detect at this temperature).

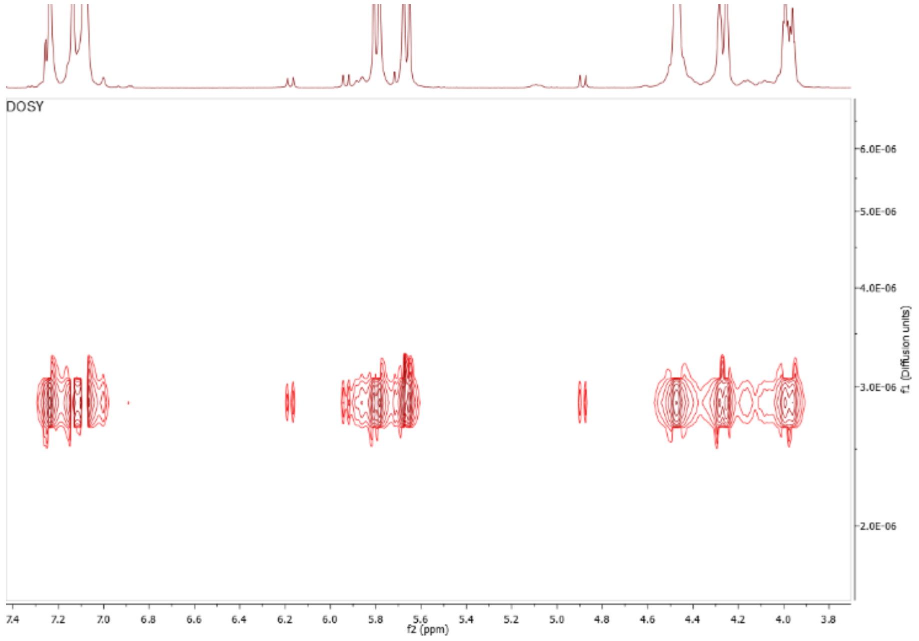

Figure S21: DOSY NMR spectrum of complex **3** in CD_3CN at 238 K, showing three isomers with diffusion coefficients 3.016×10^{-10} m^2s^{-1}

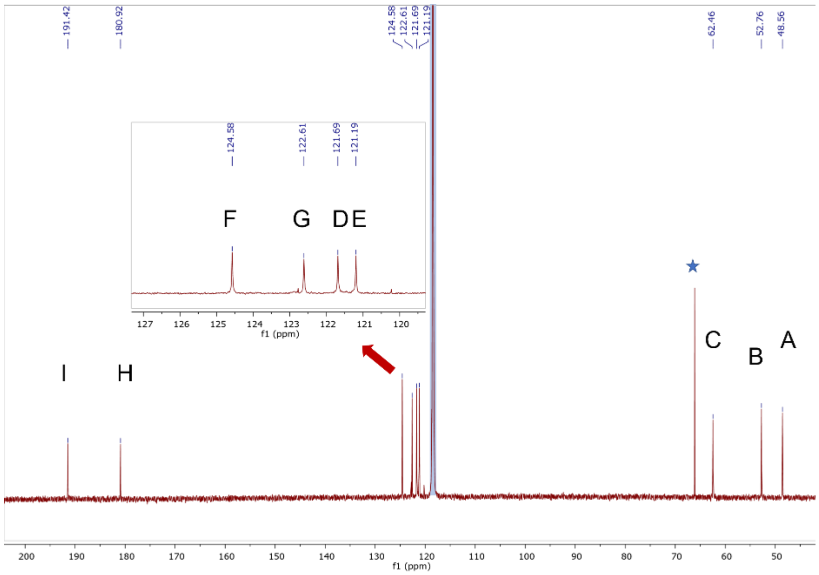

Figure S22: ^{13}C NMR spectrum of complex **3** in CD_3CN at 238 K; the inset shows a magnification of the signals of the imidazole-2-ylidene carbon atoms (the blue shaded signals correspond to the solvent peaks; the origin of the peak marked with an asterisk is unknown). Signals have been assigned based on all 2D NMR experiments.

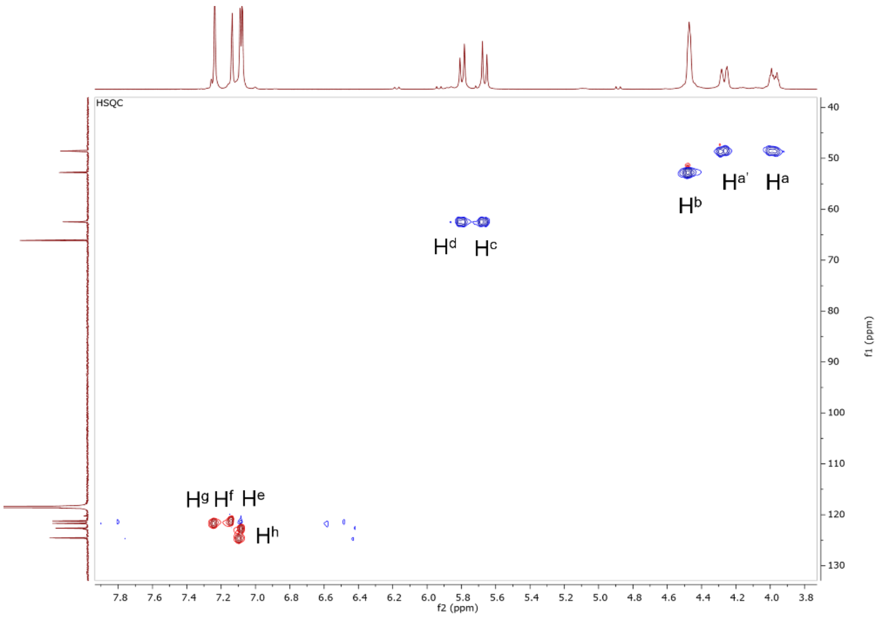

Figure S23: HSQC NMR spectrum of complex **3** in CD_3CN at 238 K. Signals have been assigned based on all 2D NMR experiments.

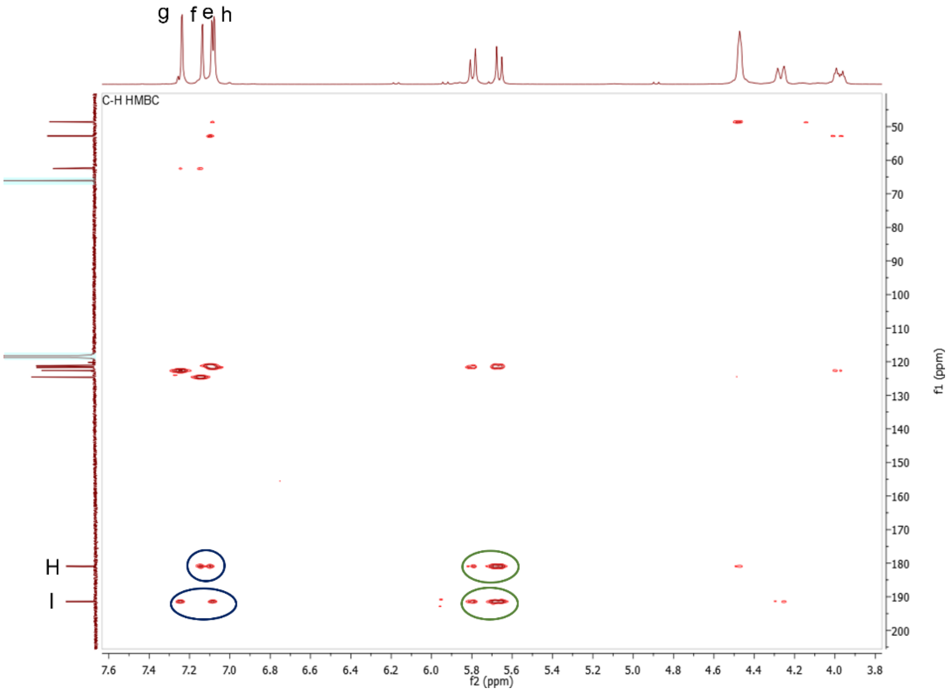

Figure S24: CH HMBC spectrum of complex **3** in CD_3CN at 238 K. The blue rounds are showing the correlation between the H_e, H_f with 'H' carbon and H_h, H_g with 'I' carbon.

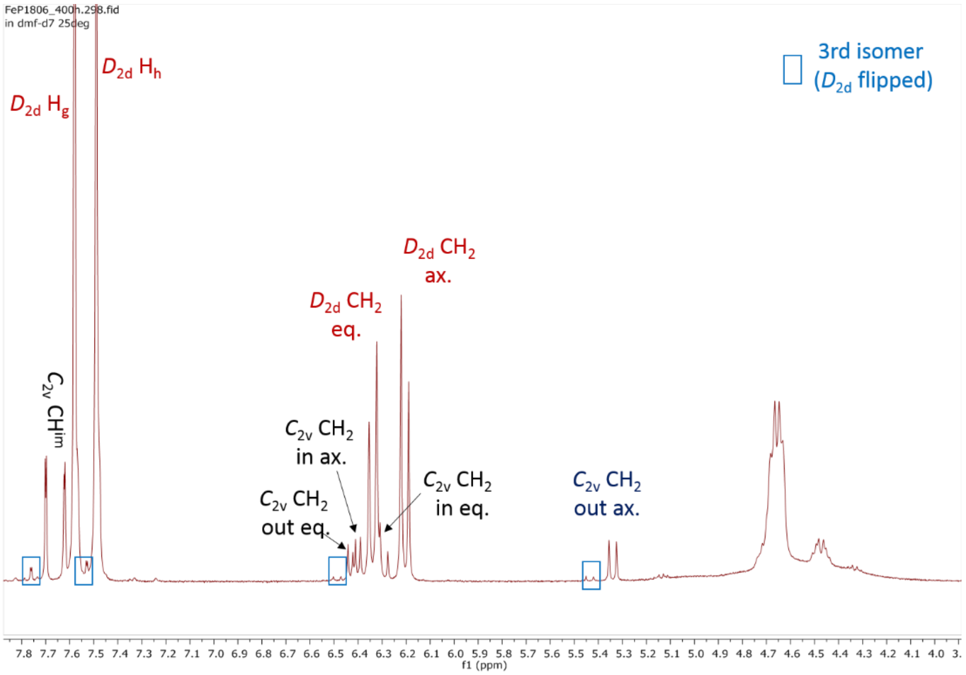

Figure S25: ^1H NMR spectrum of complex **3** in DMF-d_7 at 298 K. The assignment of peaks is based on the suite of 2D NMR spectra.

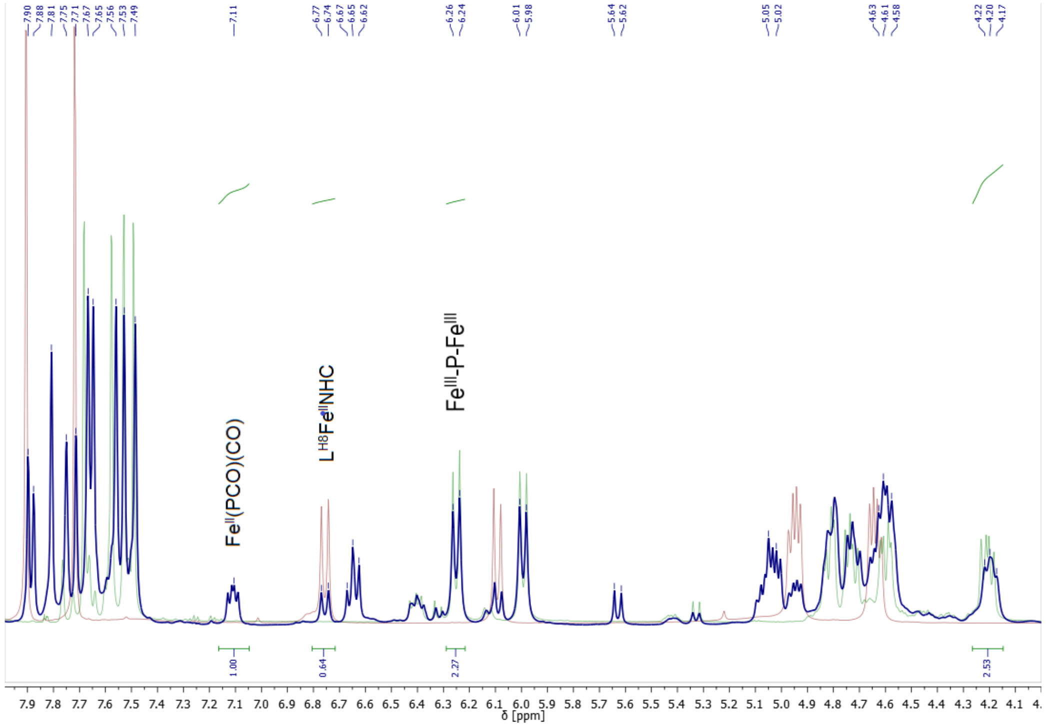

Figure S26: ^{1}H NMR spectrum of the reaction mixture after combining **1** and 0.6 eq. of Na(OCP) in DMF-d_7 at 238 K. Integration shows 1:1 formation of **2** and **3**, where >50% complex **1** is still in the solution (blue line shows the reaction mixture, red is for genuine **1** and green for genuine **3**).

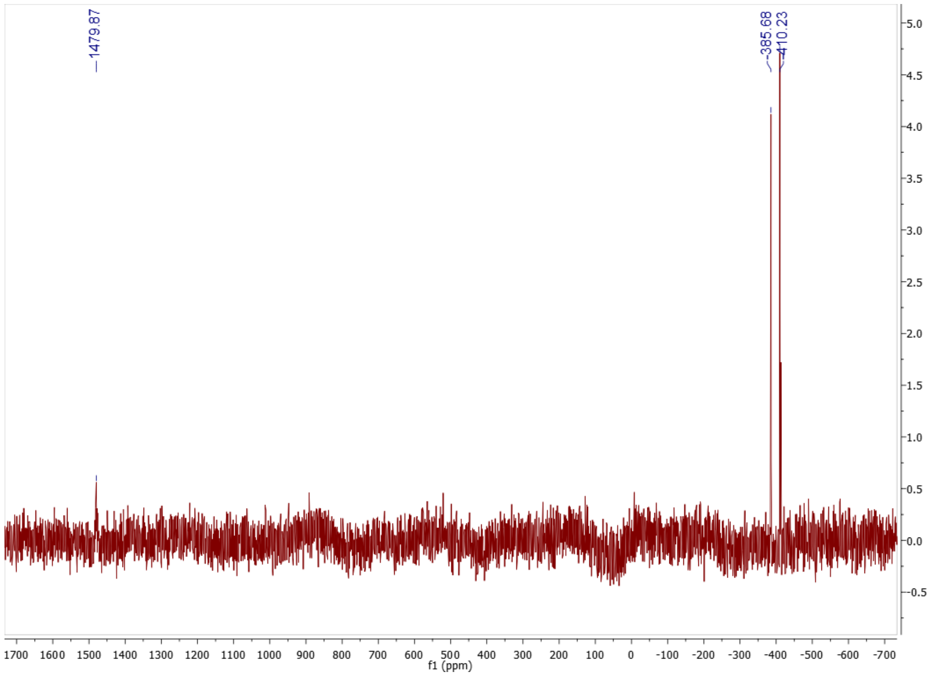

Figure S27: ^{31}P NMR spectrum of the reaction mixture after combining **1** and 0.6 eq. of Na(OCP) in DMF-d_7 at 238 K, showing signals for **2** (-410 ppm), **3** (+1477.9 ppm) and Na(OCP) (-385.6 ppm).

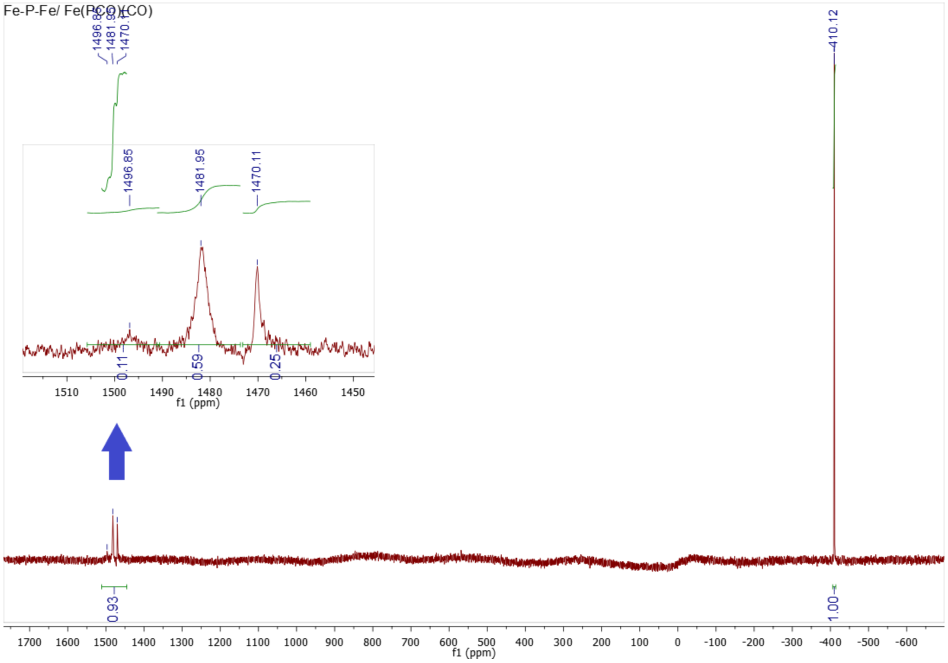

Figure S28: ^{31}P NMR spectrum of the reaction mixture after completion of the reaction, showing the signals for **2** and **3** in DMF-d_7 at 298 K. The inset shows a magnification of the low-field region with resonances for the three isomers of

4. NMR Spectra for 4

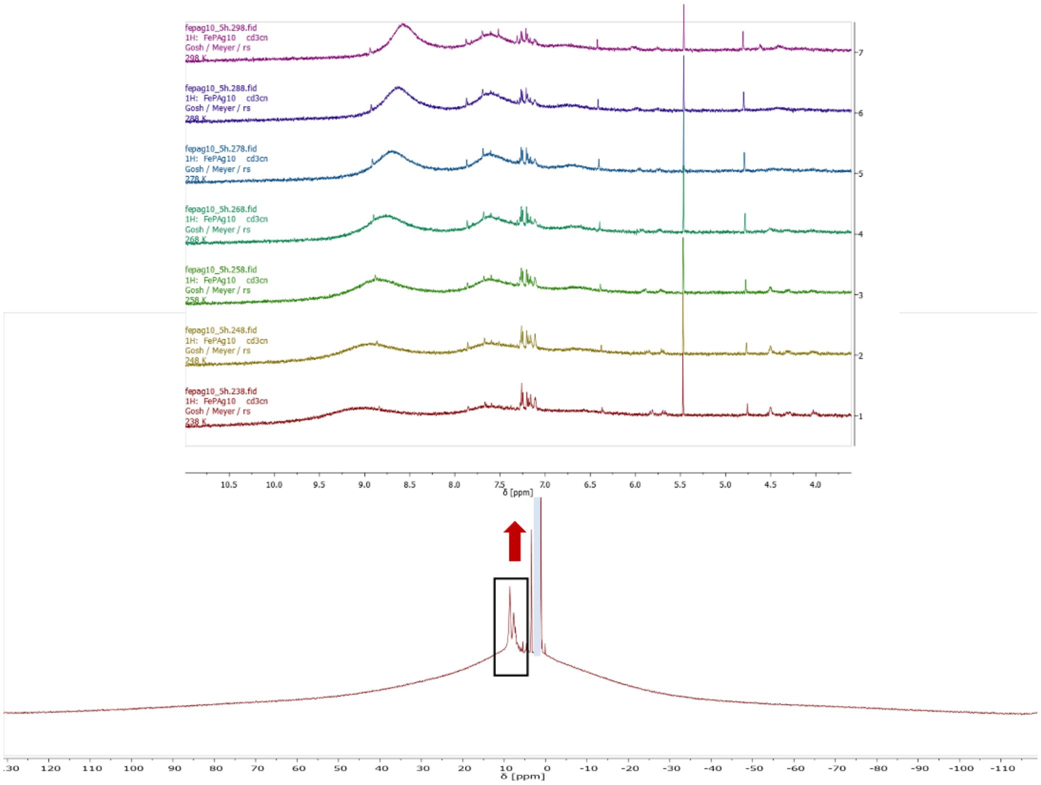

Figure S29. Variable temperature ^{1}H NMR spectrum of paramagnetic complex **4** in the temperature range from -35 ^{o}C to +25 ^{o}C.

5. NMR Spectra for 5

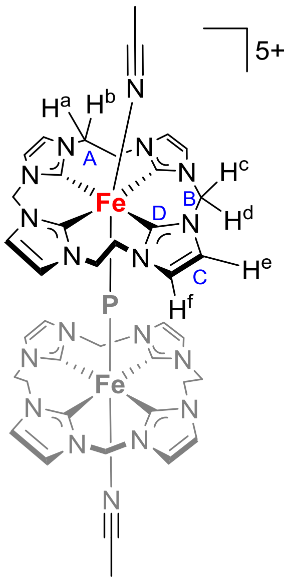

Numbering scheme for **5** (for NMR assignment)

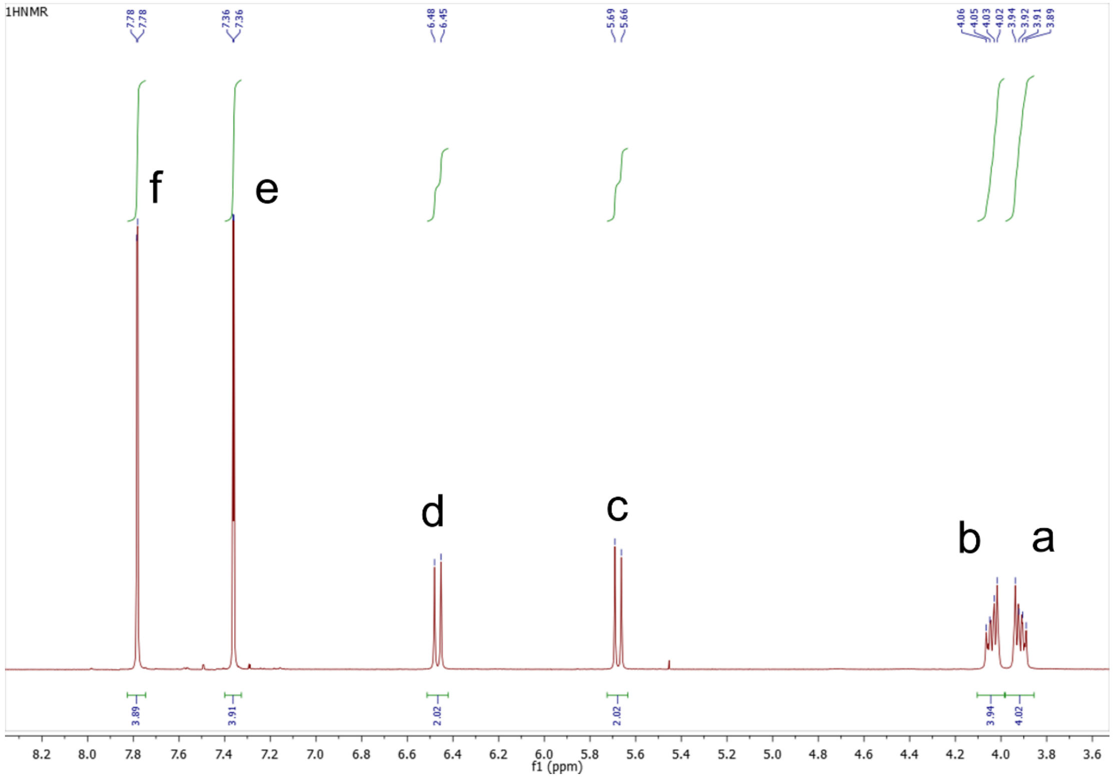

Figure S30. ^{1}H NMR spectrum of complex **5** at 238 K in CD_3CN.

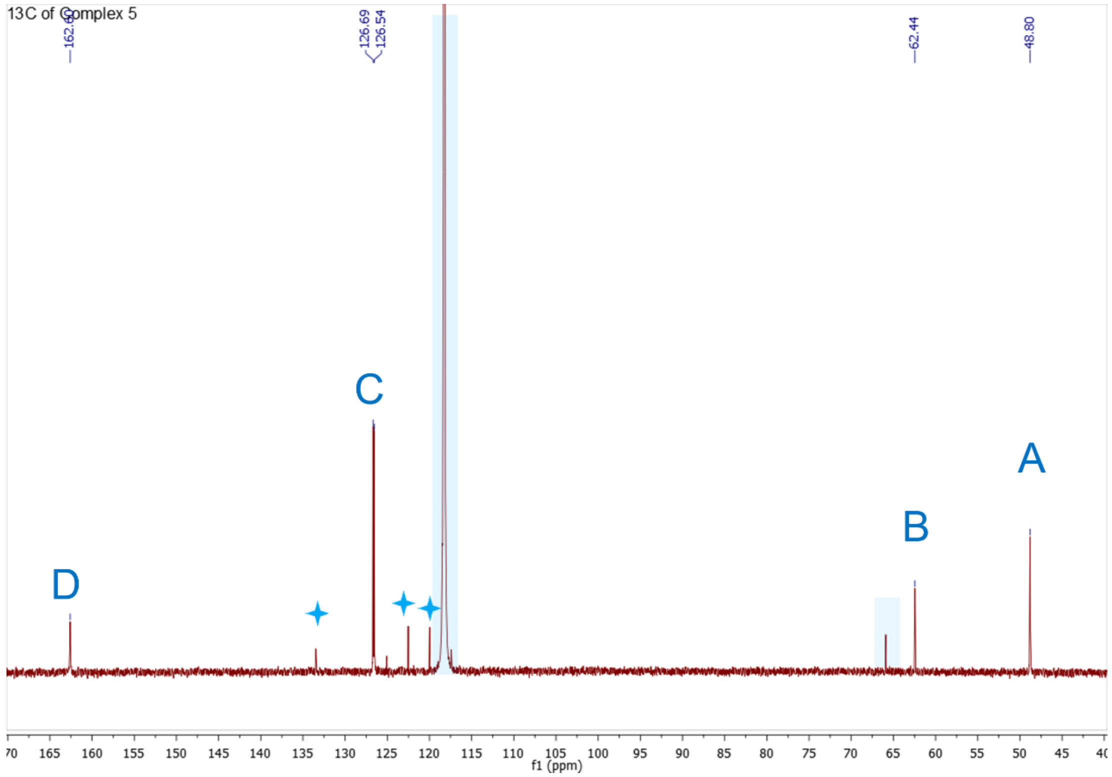

Figure S31. ^{13}C NMR spectrum of complex **5** at 238 K in CD_3CN. The peak labelled with asterisks have not been assigned (the blue shaded signals correspond to the solvent peaks).

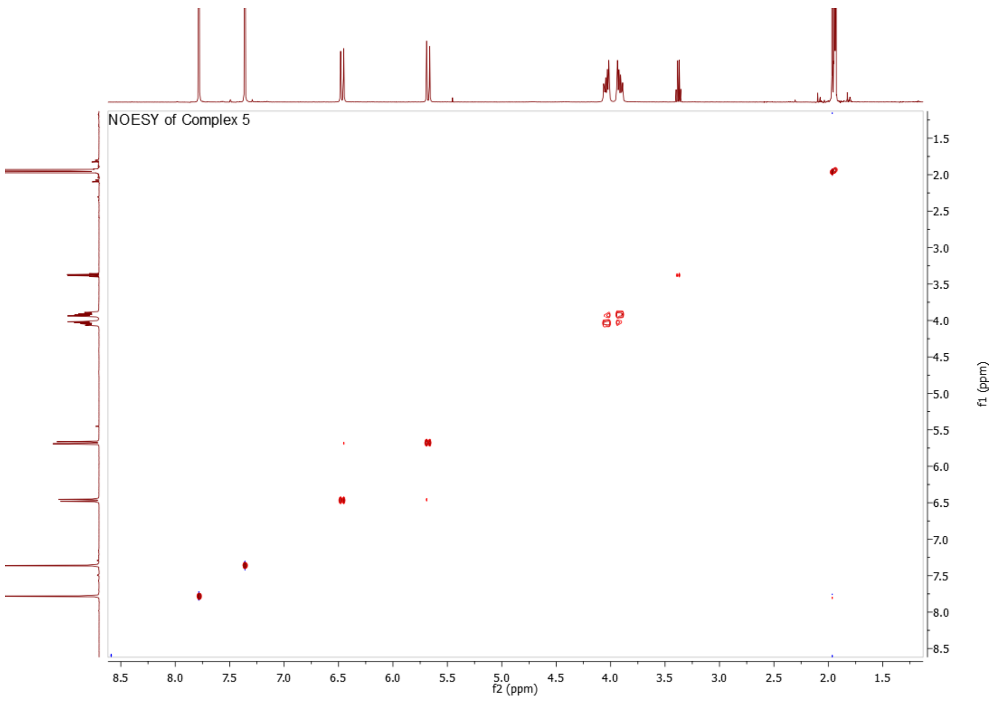

Figure S32. NOESY NMR spectrum of complex **5** at 238 K in CD_3CN.

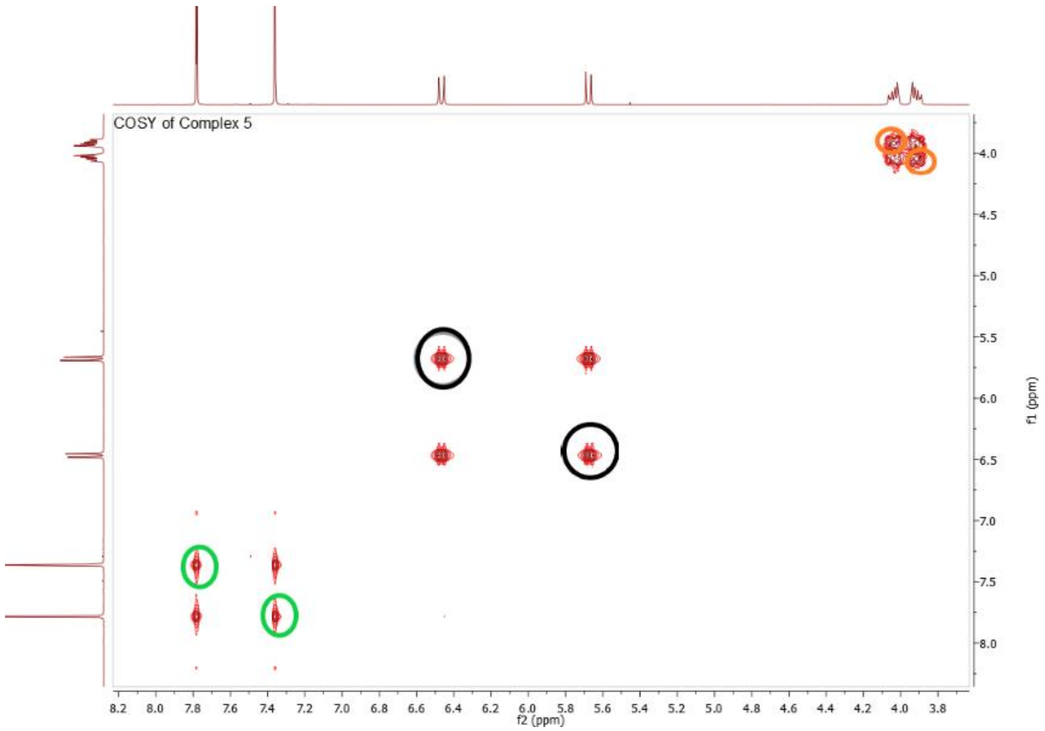

Figure S33. COSY NMR spectrum of complex **5** at 238 K in CD_3CN; orange circles for $-CH_2CH_2$, black circles for $-CH_2$ and green circles for imidazole-2-ylidene protons.

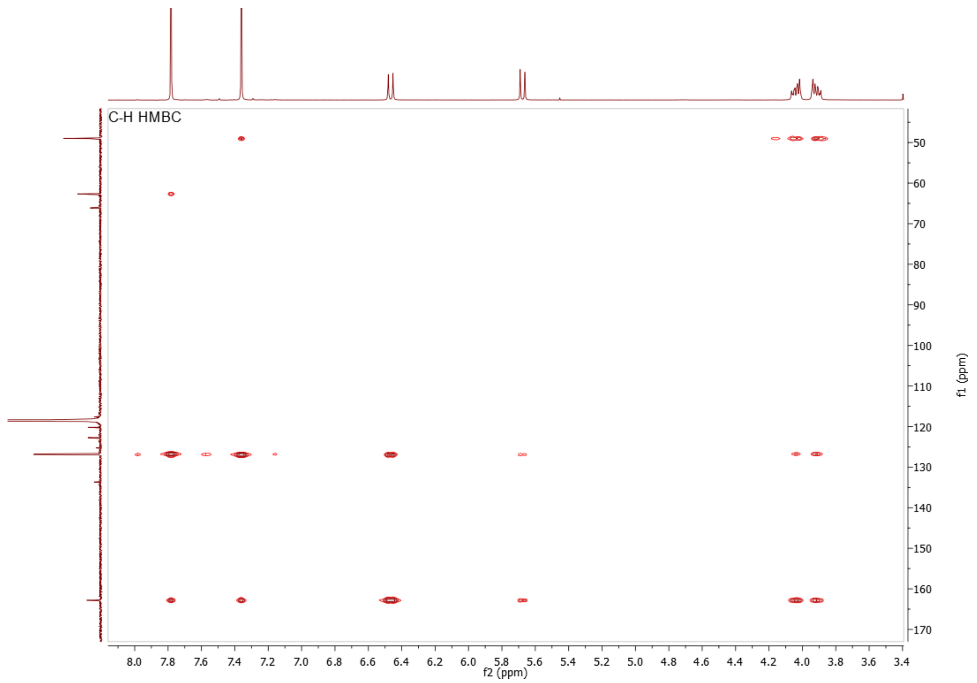

Figure S34. CH HMBC NMR spectrum of complex **5** at 238 K in CD_3CN.

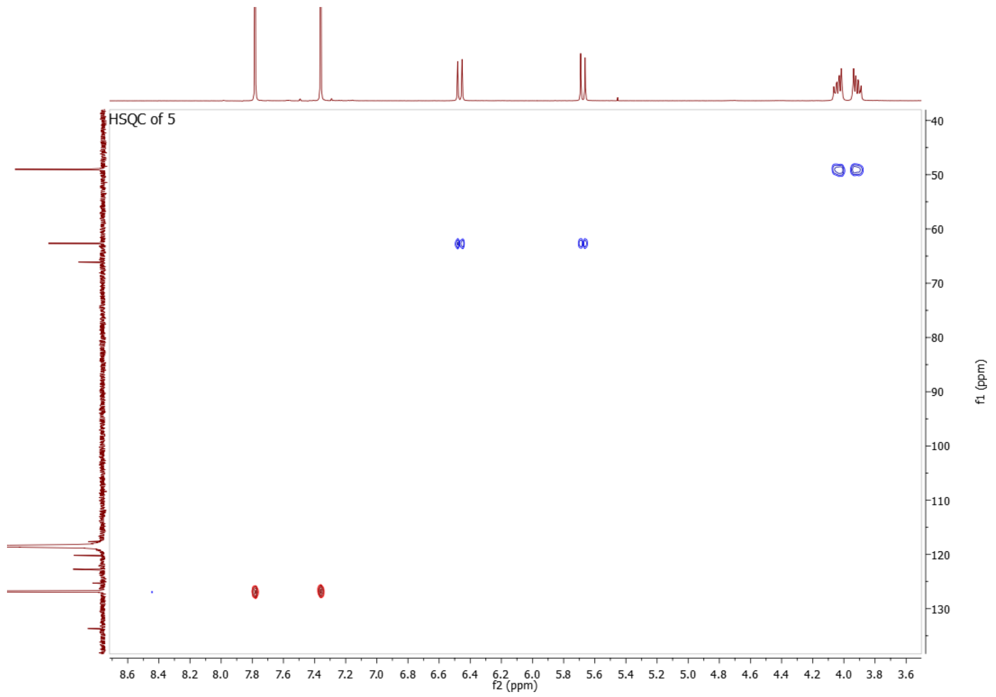

Figure S35. HSQC NMR spectrum of complex **5** at 238 K in CD_3CN.

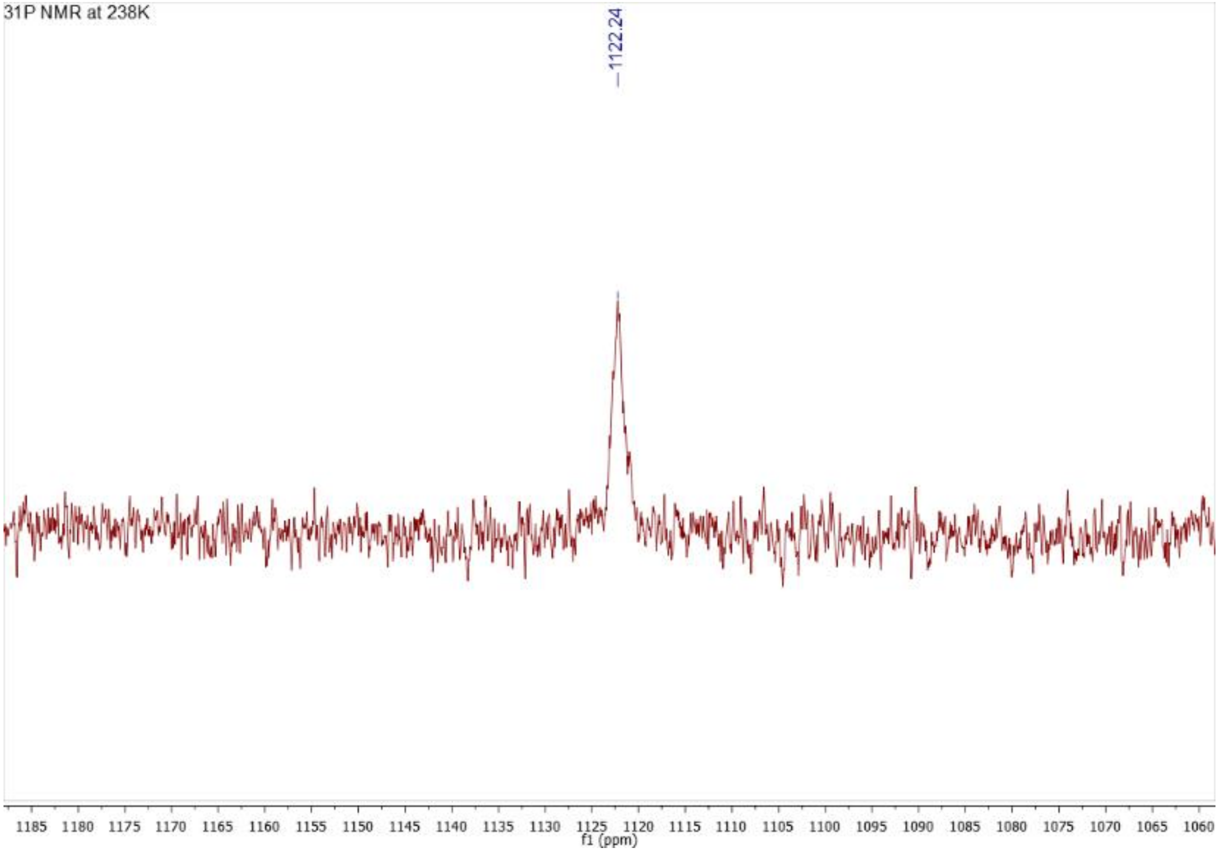

Figure S36. ^{31}P NMR spectrum of complex **5** at 238 K in CD_3CN.

6. IR Spectra

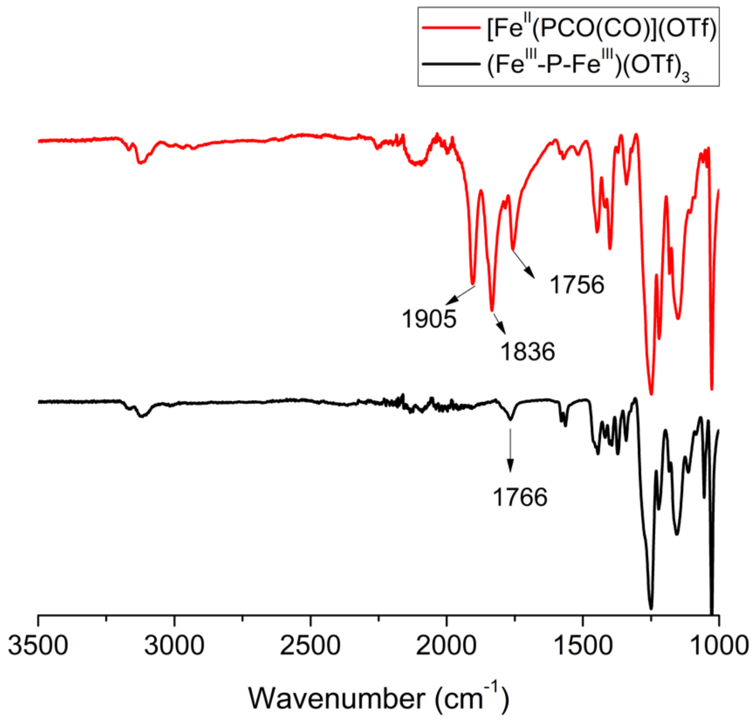

Figure S37. ATR-IR spectra of complex **2** (red) and complex **3** (black).

7. UV-vis Spectra

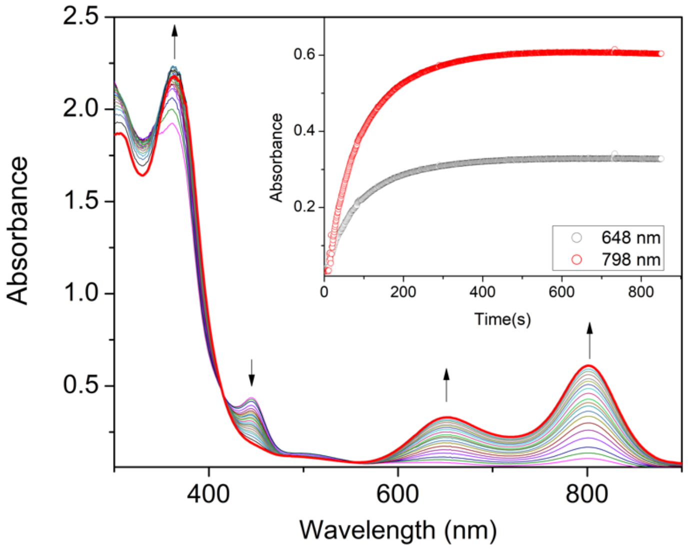

Figure S38: UV-vis monitoring of the reaction of **1** and 0.6 eq. of Na(OCP) in DMF at -35 ^{o}C. The inset shows time traces for the evolution of the bands with λ_{max} = 798 and 648 nm characteristic for **3**.

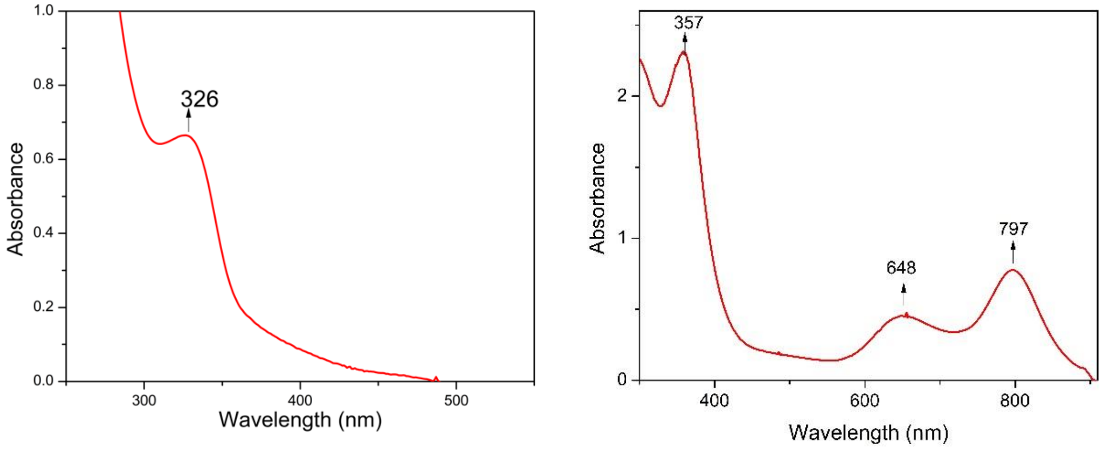

Figure S39. UV-vis spectra of **2** (left) and **3** (right) in CH_3CN at 298 K.

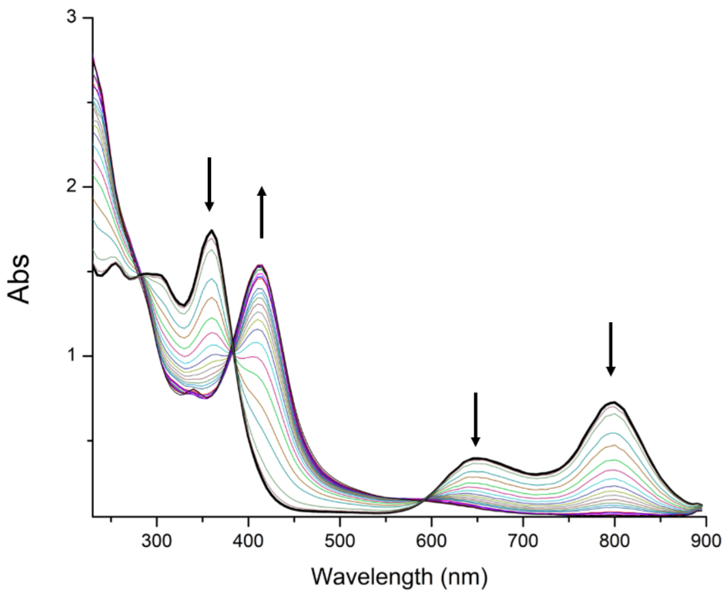

Figure S40. UV-vis monitoring of the reaction of **3** with 1 equiv. AgOTf in CH_3CN to give **4**.

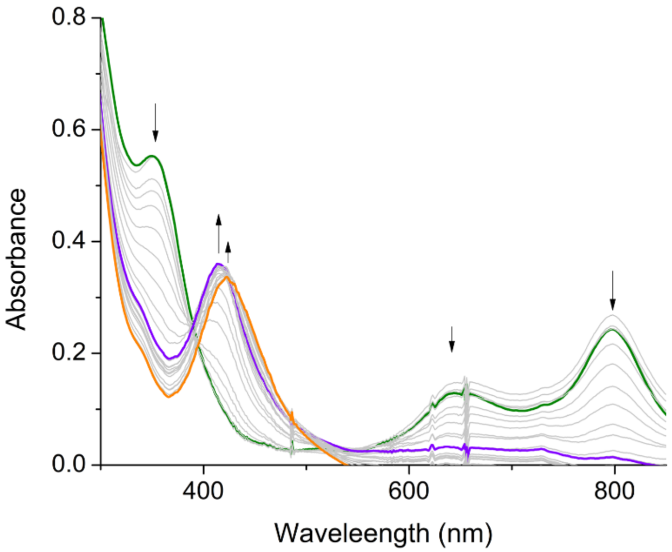

Figure S41. UV-vis monitoring of the reaction of **3** with 2.5 equiv. AgOTf in CH_3CN to give **4** (blue spectra) and then subsequently formation of **5** (orange spectra).

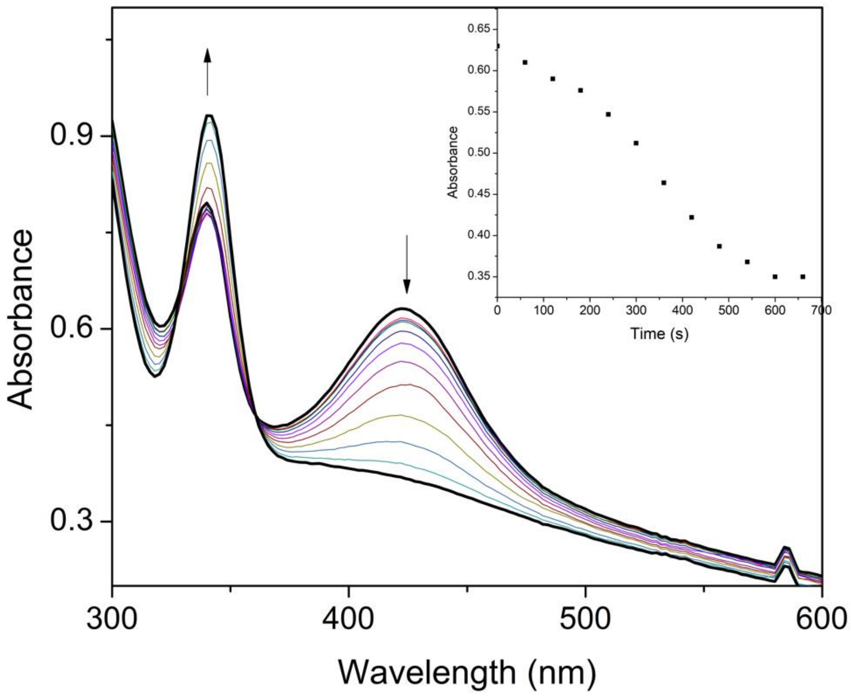

Figure S42. Degradation of complex **5** in CH_3CN at rt (degradation starts at -10 ºC; the inset shows the time trace for the evolution of the band with λ_{max} = 424 nm.

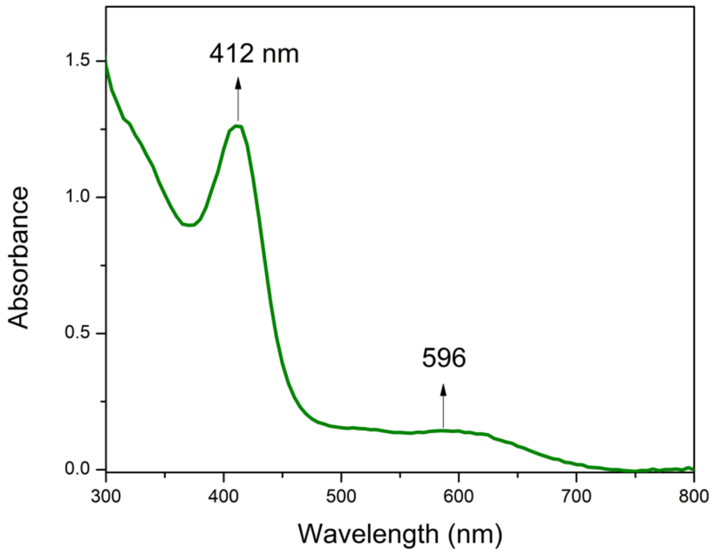

Figure S43. UV-vis spectrum of complex **4** in CH_3CN at 298 K.

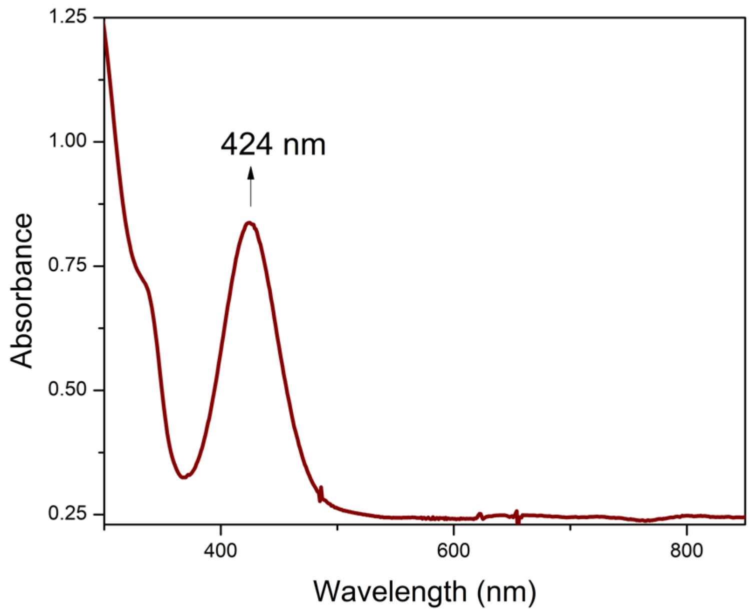

Figure S44. UV-vis spectrum of complex **5** in CH_3CN at 238 K.

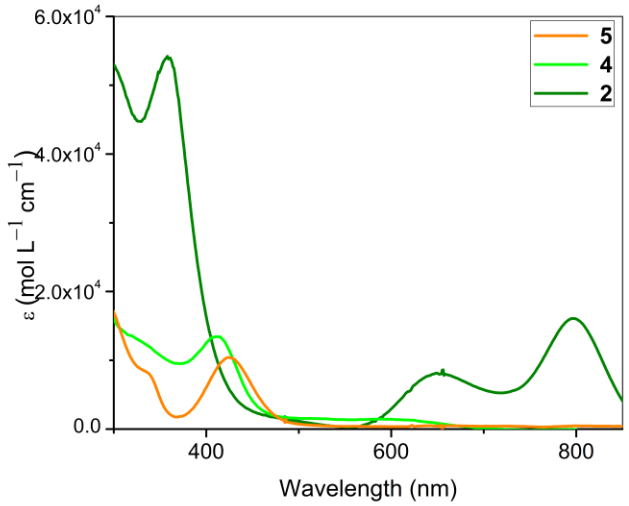

Figure S45. Overlay of the UV-vis spectra of complexes **3 – 5** in MeCN.

8. ESI Mass Spectrometry

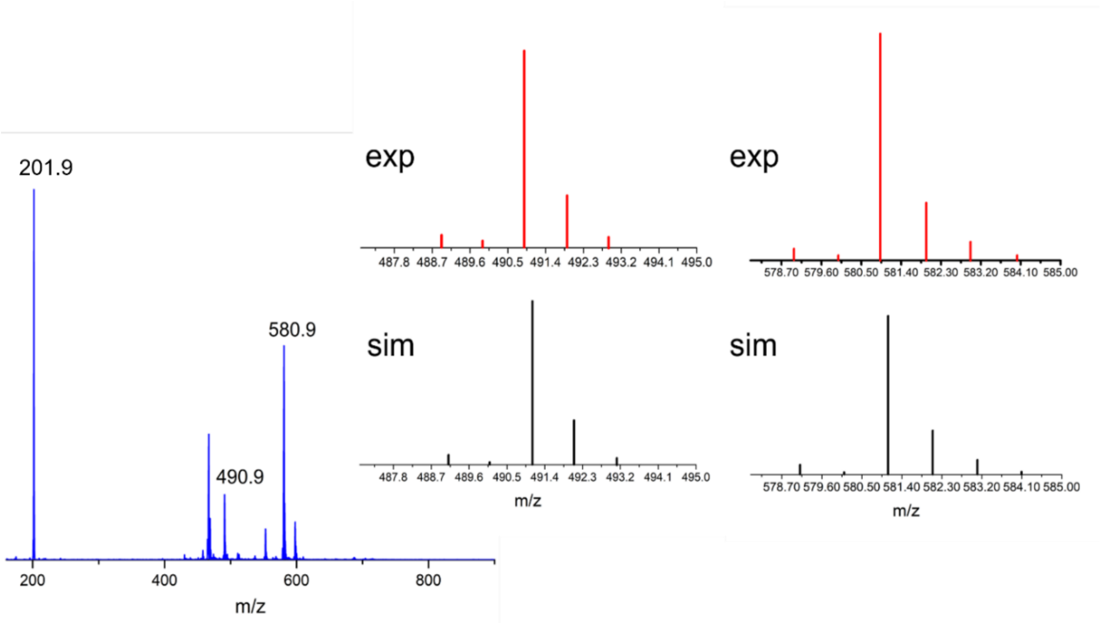

Figure S46. ESI(+) mass spectrum of a CH_3CN solution of **2** and experimental (red) and simulated (black) isotopic distribution for the peak patterns around *m*/*z* = 490.9 ($[LFe(PCO)(CO)]^+$) and 580.9 ($[LFe(CO)(OTf)]^+$).

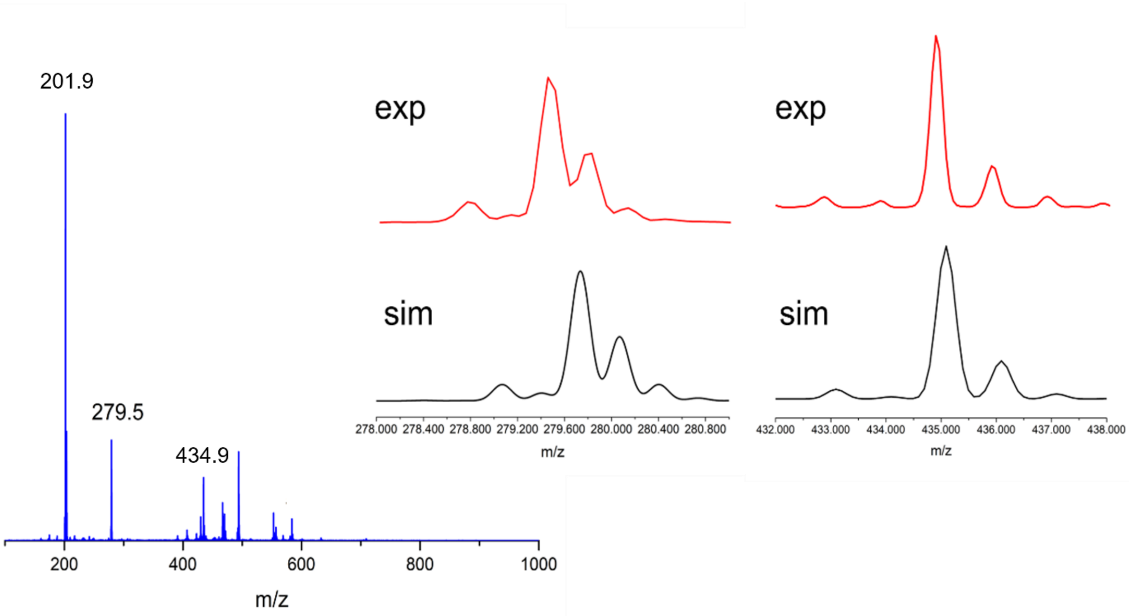

Figure S47. ESI(+) mass spectrum of a CH_3CN solution of **3** and experimental (red) and simulated (black) isotopic distribution for the peak patterns around *m*/*z* = 279.5 and ($[LFePFeL]^{3+}$), and 434.9 ($[LFeP]^+$).

9. Magnetic Susceptibility Measurements

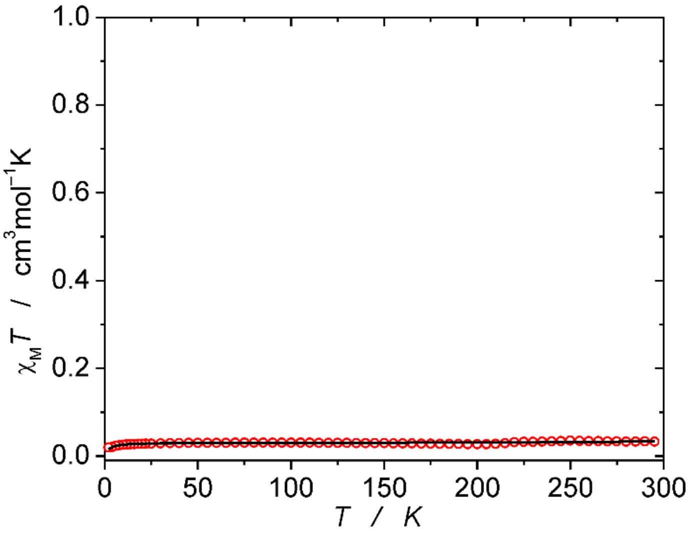

Figure S48: $\chi_M T$ versus T plot for **3**. Solid line represents the calculated curve fit for the $S_1 = S_2 = ½$ system with parameters: $g_1 = g_2 = 2.00$ (fixed), $J = –600\ cm^{-1}$ (fixed), $PI = 0.7$ % (with $S = 5/2$) and $TIP = 450 \cdot 10^{-6}\ cm^3mol^{-1}$.

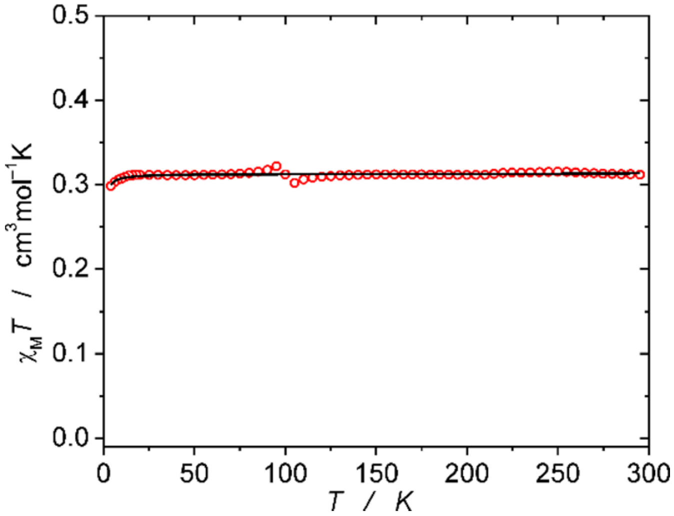

Figure S49: $\chi_M T$ versus T plot for **4**. Solid lines represent the calculated curve fits with parameters: $g_1 = 2.00$ (fixed), $g_2 = 1.87$, $J = –600\ cm^{-1}$ (fixed) and $TIP = 480 \cdot 10^{-6}\ cm^3mol^{-1}$ (using $S_1 = ½$ and $S_2 = 1$).

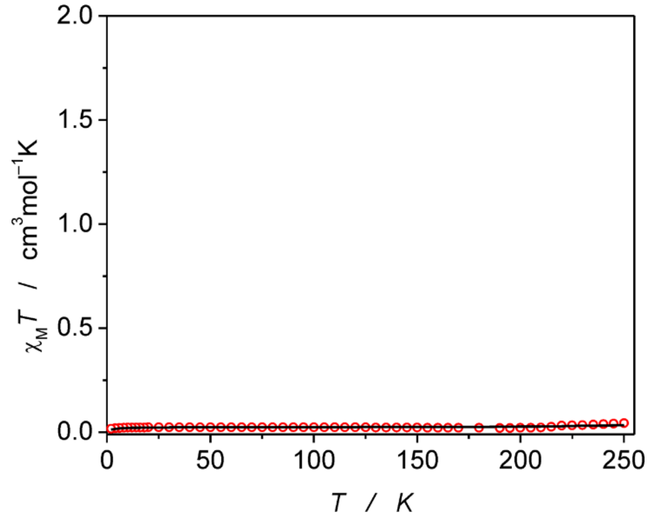

Figure S50: $\chi_M T$ versus T plot for **5**. Solid lines represent the calculated curve fits with parameters: $g_1 = g_2 = 2.00$ (fixed), $J = -500$ cm^{-1} (fixed), $PI = 0.6$ % (with $S = 5/2$) and $TIP = 540 \cdot 10^{-6}$ cm^{3}mol^{-1} (using $S_1 = S_2 = 1$).

10. EPR Spectra

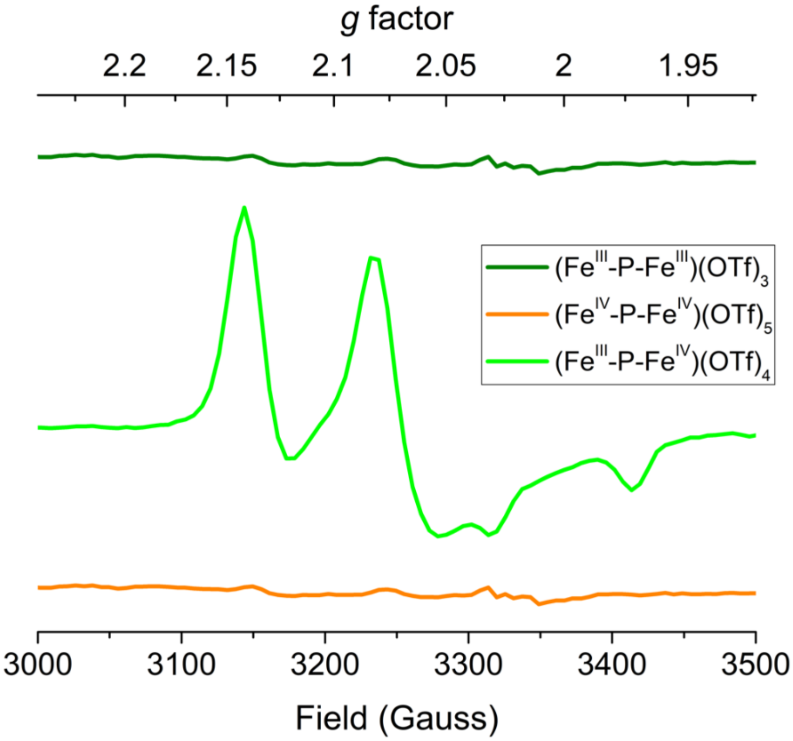

Figure S51. EPR spectra of complexes **3** – **5** at 153 K in frozen CH_3CN; **3** and **5** are EPR silent.

11. (Spectro)Electrochemical Measurements

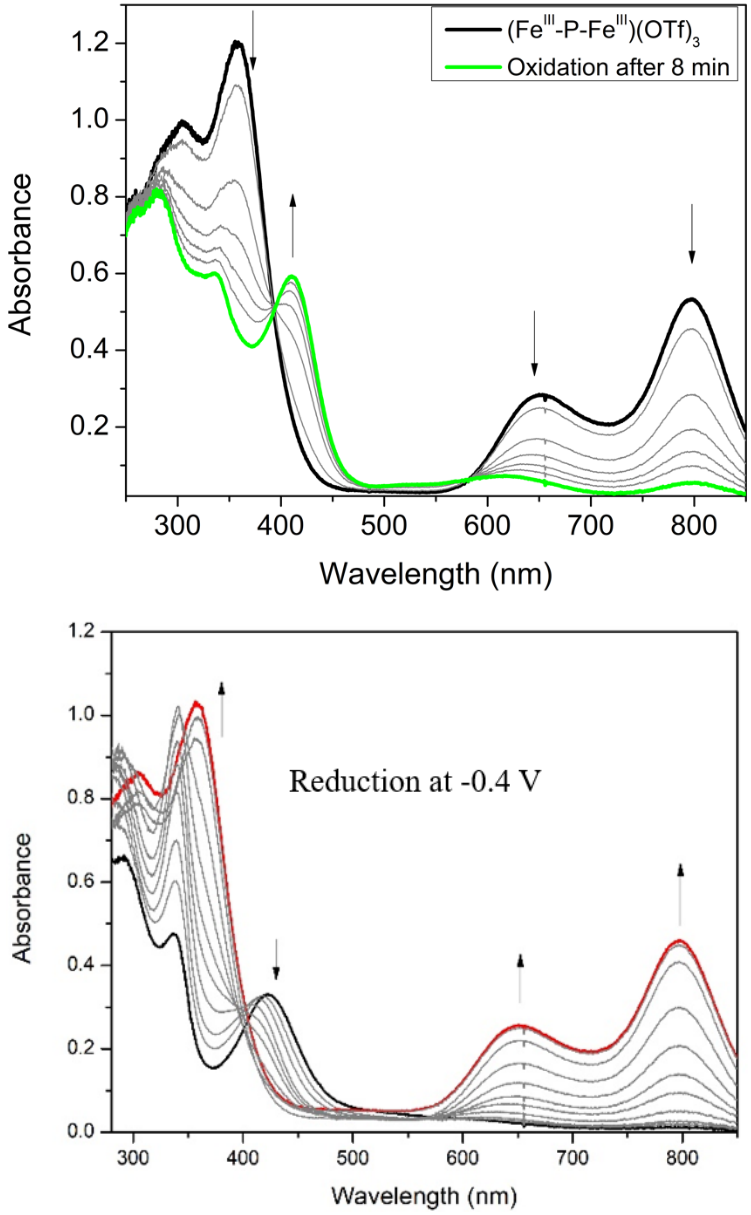

Figure S52. Spectroelectrochemical monitoring of the oxidation of **3** in CH_3CN at -0.1 V (vs Fc/Fc^+) during the first 8 min to give **4** (top) and of the re-reduction after applying a potential of -1.0 V (vs Fc/Fc^+) (bottom) (-0.4 V vs Ag).

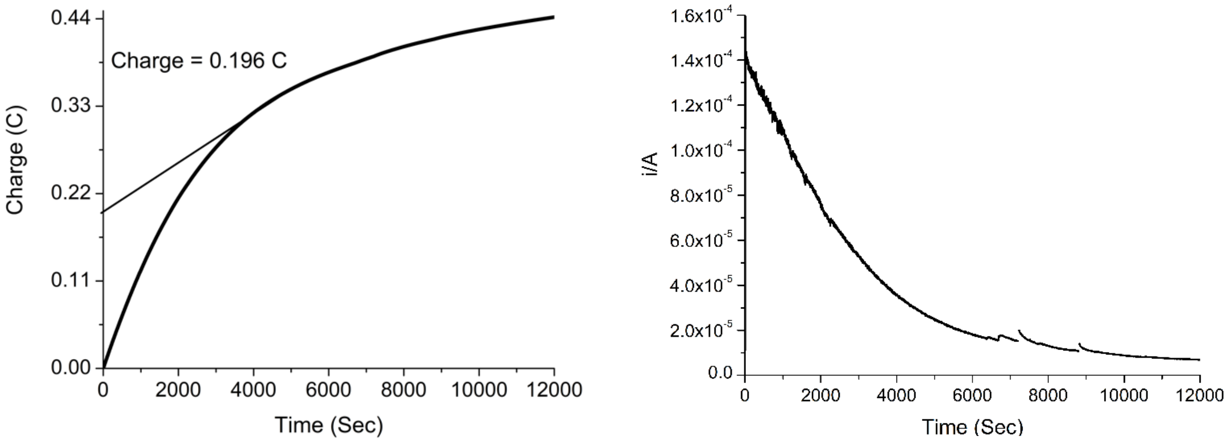

Figure S53. Bulk electrolysis of complex **3** in CH_3CN/0.1 M $[N^nBu_4]PF_6$ at rt; 1.3 mg (1.01 mmol) of **3** was taken for the experiments. Working electrode Pt mesh. E applied at -0.1 V (vs Fc/Fc^+).

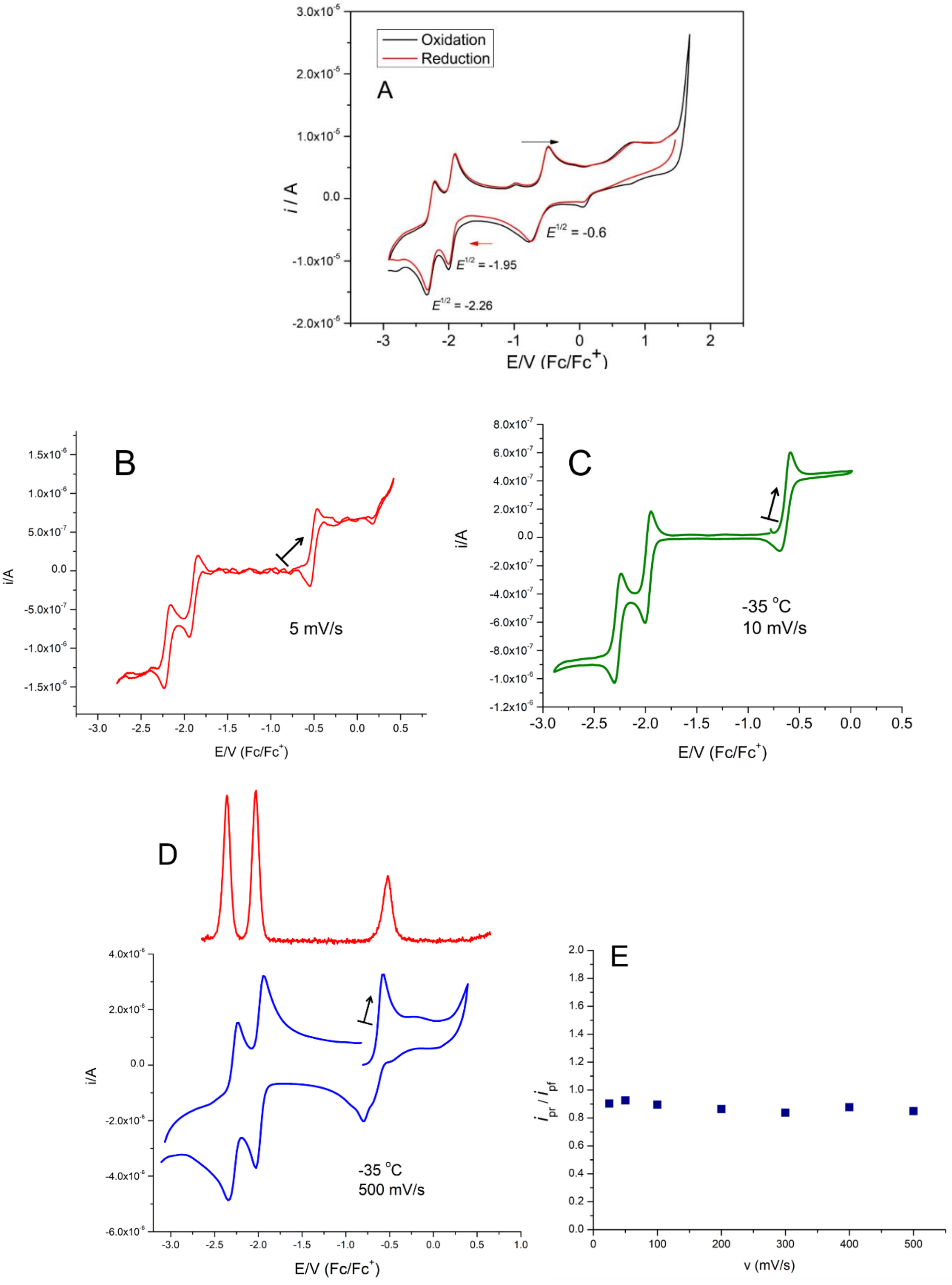

Figure S54. **A**: Cyclic voltammograms (from +2 to -2.5V and reverse) of **3** in CH_3CN/0.1 M [$^{n}Bu_4N$]PF_6 at rt at 200 mV/s scan rate. **B**: CV at 5 mV/s scan rate at rt. **C**: CV at -35 ºC at 10 mV/s scan rate. **D**: CV at -35 ºC at 500 mV/s scan rate, also the SWV at the same condition. **E**: i_{pr}/i_{pf} versus scan rate v for the process around -0.6 V.

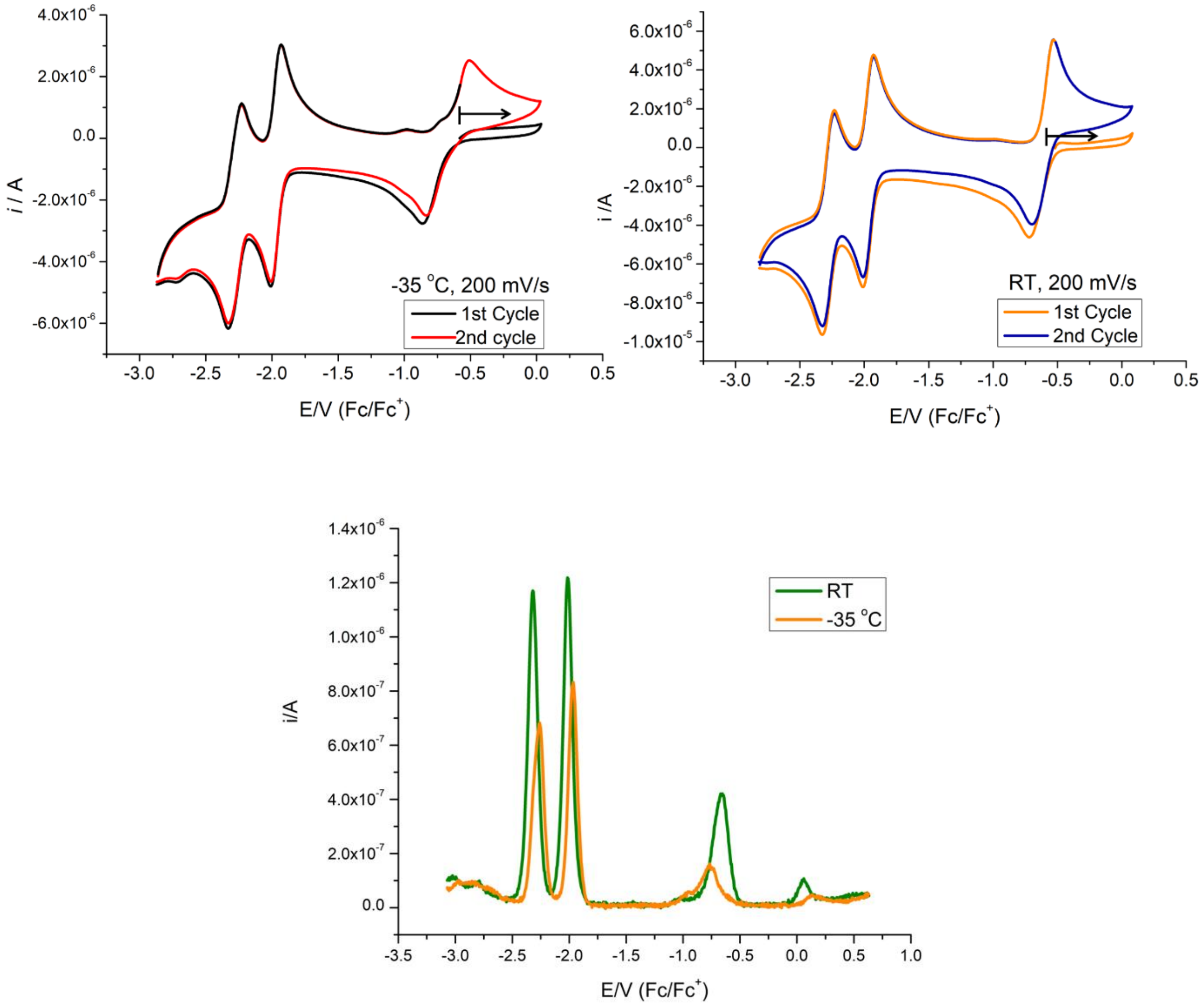

Figure S55. CV of complex **4** at -35 °C (200 mV/s) (top left) and at rt (top right) and SWVs of **4** at -35 °C and rt (bottom).

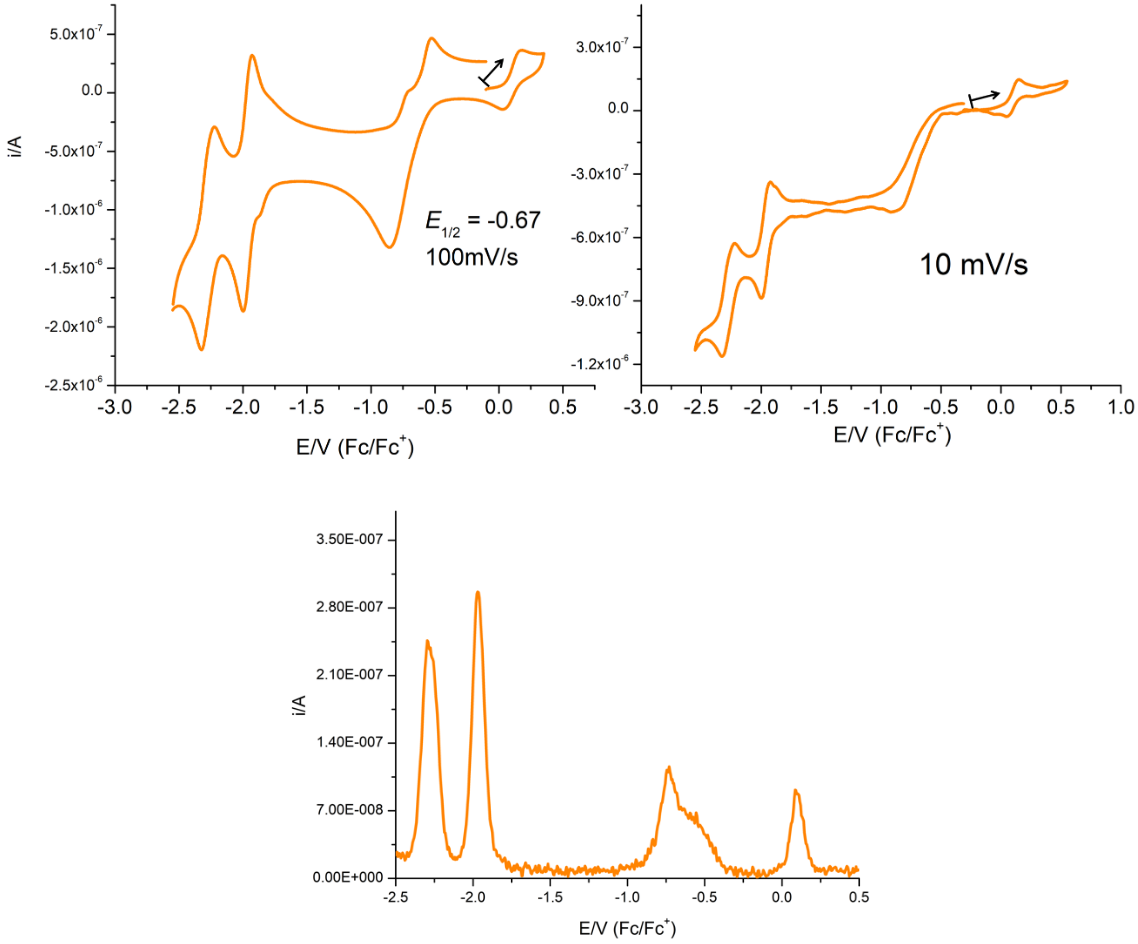

Figure S56. CV of complex **5** at -35 ºC at 100 mV/s scan rate (top left) and at 10 mV/s (top right) and SWVs of **5** (bottom) showing a double hump at around -0.6 V.

12. X-ray Crystallography

Crystal data and details of the data collections are given in Table S1, molecular structures are shown in Figures S48 – S51. X-ray data were collected on a STOE IPDS II diffractometer (graphite monochromated Mo-Kα radiation, λ = 0.71073 Å) by use of w scans at –140 °C. The structures were solved with SHELXT and refined on F^2 using all reflections with SHELXL-2018.[7] Non-hydrogen atoms were refined anisotropically. Hydrogen atoms were placed in calculated positions and assigned to an isotropic displacement parameter of 1.5/1.2 U_{eq}(C). Crystals of **3** were found to be twinned (twin law: 1 0.021 0.003, 0 –1 0, 0 0 –1; BASF: 0.126(2)). Acetonitrile molecules and $CF_3SO_3^-$ anions were found to be disordered in case of **5** (occupancy factors: MeCN = 0.543(18)/0.457(18) & 0.508(6)/0.492(6); $CF_3SO_3^-$ = 0.9319(18)/0.0681(18), 0.536(5)/0.464(5) & 0.548(4)/ 0.452(4)) and were refined using SAME and RIGU restraints and EADP constraints. In case of **4** only the cationic part could be identified from the crystal structure (monoclinic, *C*2/*c*, *a*=42.62 Å, *b*=15.75 Å, *c*=23.15 Å, β=119°). The quality of the diffraction data, however, was not sufficient for anisotropic refinement or discussion of bonding parameters. Face-indexed absorption corrections were performed numerically with the program X-RED.[8]

Table S1**.** Crystal data and refinement details for **2**, **3**, and **5**.

**compound**	**2 (mmg23)**	**3 (mmg21)**	**5 (mmg27)**
empirical formula	$C_{21}H_{20}F_3FeN_8O_5PS$	$C_{49}H_{55}F_9Fe_2N_{21}O_9PS_3$	$C_{53}H_{58}F_{15}Fe_2N_{22}O_{15}PS_5$
moiety formula	$C_{20}H_{20}FeN_8O_2P^+$, $CF_3O_3S^-$	$C_{36}H_{40}Fe_2N_{16}P^{3+}$, 3($CF_3O_3S^-$), 5($C_2H_3N$)	$C_{40}H_{46}Fe_2N_{18}P^{5+}$, 5($CF_3O_3S^-$), 4($C_2H_3N$)
formula weight	640.33	1491.99	1831.18
crystal size [mm³]	0.34 x 0.18 x 0.11	0.50 x 0.22 x 0.11	0.50 x 0.48 x 0.28
crystal system	triclinic	triclinic	triclinic
space group	*P*–1 (No. 2)	*P*–1 (No. 2)	*P*–1 (No. 2)
a [Å]	9.0146(6)	9.2077(3)	12.8660(3)
b [Å]	12.2884(8)	14.7582(5)	12.8759(3)
c [Å]	12.7514(8)	22.9930(8)	23.3195(6)
α [°]	107.576(5)	95.053(3)	81.322(2)
β [°]	107.231(5)	90.104(3)	85.362(2)
γ [°]	102.000(5)	90.948(3)	70.803(2)
V [Å³]	1215.64(15)	3111.91(18)	3604.54(16)
Z	2	2	2
ρ [g·cm⁻³]	1.749	1.592	1.687
F(000)	652	1528	1864
μ [mm⁻¹]	0.849	0.691	0.687
T_{min} / T_{max}	0.4452 / 0.7158	0.7997 / 0.9413	0.7780 / 0.9704
θ-range [°]	1.815 - 26.831	1.385 - 25.803	1.677 - 26.870

hkl-range	±11, ±15, ±16	–11 to 9, ±17, ±27	±16, –15 to 16, ±29
measured refl.	16042	40161	49368
unique refl. [R_{int}]	5168 [0.0801]	11712 [0.0879]	15212 [0.0357]
observed refl. ($I > 2\sigma(I)$)	3701	9575	12783
data / restr. / param.	5168 / 0 / 361	11712 / 0 / 854	15212 / 753 / 1253
goodness-of-fit (F^2)	1.049	1.038	1.023
$R1$, $wR2$ ($I > 2\sigma(I)$)	0.0571 / 0.1460	0.0913 / 0.2329	0.0453 / 0.1112
$R1$, $wR2$ (all data)	0.0878 / 0.1655	0.1122 / 0.2557	0.0562 / 0.1188
res. el. dens. [e·Å^{-3}]	–0.470 / 1.249	–1.110 / 1.994	–0.728 / 1.177

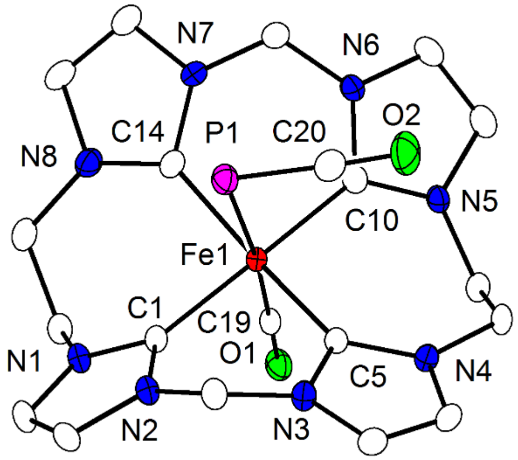

Figure S57. Plot (30% probability thermal ellipsoids) of the molecular structure of the cationic part of **2** (hydrogen atoms omitted for clarity). Selected bond lengths [Å] and angles [°]: Fe1–C10 1.965(4), Fe1–C1 1.967(4), Fe1–C5 2.007(4), Fe1–C14 2.046(4), Fe1–C19 1.740(4), Fe1–P1 2.4997(12), P1–C20 1.635(5), C20–O2 1.171(6), C19–O1 1.142(5); C10–Fe1–C1 178.25(16), C5–Fe1–C14 169.17(16), C19–Fe1–P1 174.73(13), C20–P1–Fe1 95.32(16), O1–C19–Fe1 177.6(4), P1–C20–O2 178.1(5).

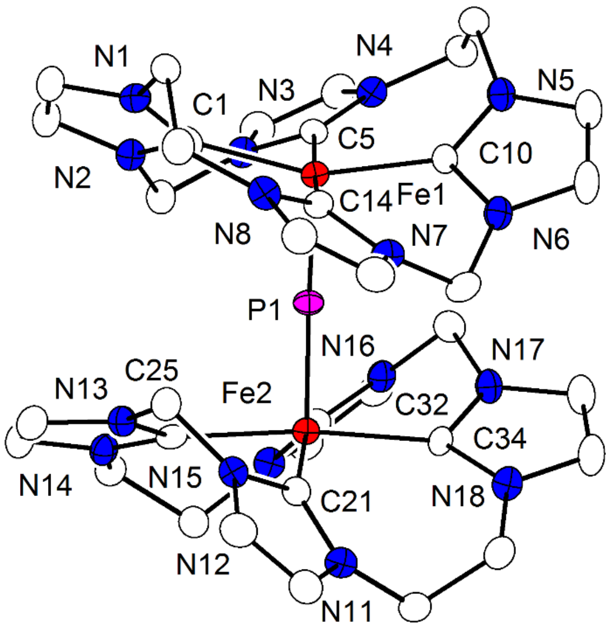

Figure S58. Plot (30% probability thermal ellipsoids) of the molecular structure of the cationic part of **3** (hydrogen atoms omitted for clarity). Selected bond lengths [Å] and angles [°]: Fe1–C1 1.953(8), Fe1–C10 1.959(7), Fe1–C14 2.007(7), Fe1–C5 2.021(7), Fe1–P1 1.993(2), Fe2–C30 1.951(7), Fe2–C25 2.006(8), Fe2–C34 2.008(7), Fe2–P1 1.998(2); C1–Fe1–C10 157.6(3), C5–Fe1–C14 174.3(3), C30–Fe2–C21 156.5(3), C25–Fe2–C34 173.1(3), Fe1–P1–Fe2 178.25(12).

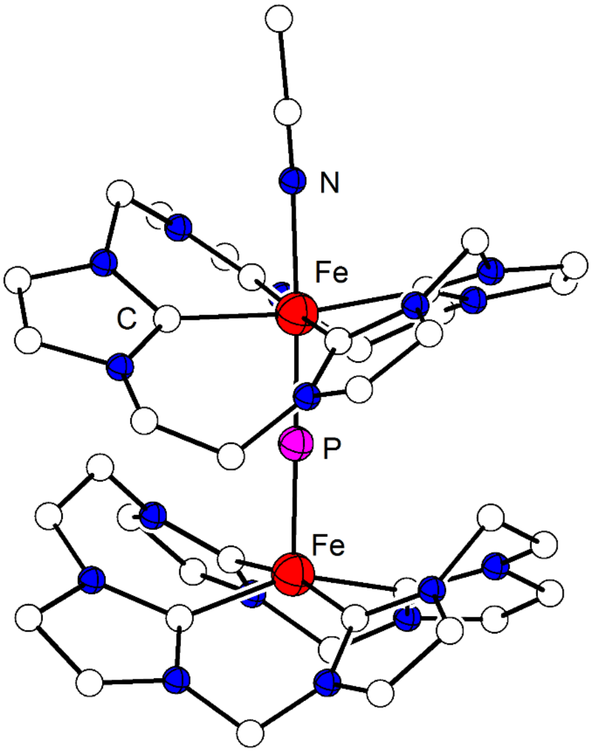

Figure S59. Plot of the molecular structure of the cationic part of **4**.

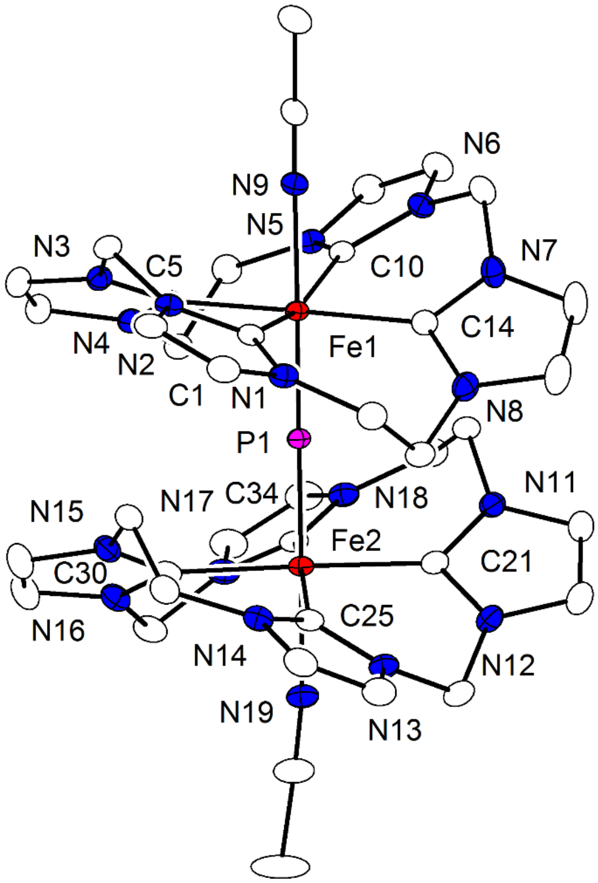

Figure S60. Plot (30% probability thermal ellipsoids) of the molecular structure of the cationic part of **5** (hydrogen atoms omitted for clarity). Selected bond lengths [Å] and angles [°]: Fe1–C14 1.994(3), Fe1–C5 1.996(2), Fe1–C1 2.046(2), Fe1–C10 2.046(2), Fe2–C21 1.993(3), Fe1–P1 2.0079(7), Fe1–N9 1.995(2), Fe2–C25 2.031(2), Fe2–C34 2.040(3), Fe2–P1 2.0016(7), Fe2–N19 2.013(2); C14–Fe1–C5 179.13(10), C1–Fe1–C10 164.55(9), N9–Fe1–P1 179.80(7), C21–Fe2–C30 178.58(10), C25–Fe2–C34 167.04(9), P1–Fe2–N19 177.36(7), Fe2–P1–Fe1 179.61(4), C19–N9–Fe1 179.3(2), C39–N19–Fe2 170.3(2).

13. DFT Calculations

Computational Setup

Geometry optimizations were performed with the B3LYP[9] density functional. The respective def2-TZVP[10] for the first coordination sphere and def2-SV(P) basis sets[11] for the remaining atoms were applied in combination with the auxiliary basis sets def2/J.[12] The RI[13] and RIJCOSX[14] approximations were used to accelerate the calculations.

The Mössbauer spectroscopic parameters were computed using the B3LYP density functional. The CP(PPP)[15] basis set for Fe and the def2-TZVP basis set for remaining atoms were used.

Isomer shifts δ were calculated from the electron densities ρ_0 at the Fe nuclei by employing the linear regression:

$$\delta = \alpha \cdot (\rho_0 - C) + \beta \qquad (1)$$

Here, C is a prefixed value, and α and β are the fit parameters. Their values for different combinations of the density functionals and basis sets can be found in our earlier work (α = –0.366, β = 2.852, C = 11810).[16]

Quadrupole splittings ΔE_Q were obtained from electric field gradients V_{ij} ($i = x, y, z;$ V_{ii} are the eigenvalues of the electric field gradient tensor) by using a nuclear quadrupole moment $Q(^{57}Fe)$ = 0.16 barn:[17]

$$DE_Q = \frac{1}{2} eQ \cdot V_{zz} \cdot \sqrt{1 + \frac{1}{3} h^2}$$

Here, $h = \frac{V_{xx} - V_{yy}}{V_{zz}}$ is the asymmetry parameter.

TDDFT calculations were also performed with B3LYP in conjunction with the def2-TZVP basis sets for all atoms.

All computations in this work were carried out with the ORCA program package.[18]

We took complex **3** as example to test closed- and open-shell singlet calculations. Our results showed that key metric parameters calculated by the restricted B3LYP calculations are in reasonable agreement with those found experimentally. In contrast, the unrestricted B3LYP computations indeed delivered an open-shell singlet solution, but the computed geometry of **3** having a substantially bent Fe-P-Fe core (159.3˚) is distinct from that determined by X-ray crystallography. Despite of this, the three non-bonding molecular orbitals in the spin-up manifold involving the Fe-d_{z2}, -d_{xz} and -d_{yz} orbitals are only slightly different from those in the spin-down set (Figure S53), and the difference can be readily attributed to the bent Fe-P-Fe core. Thus, the broken-symmetry solution is, in fact, qualitatively the same as that predicted by the restricted

computations. On the basis of these findings, we did not employ broken-symmetry calculations further for complexes **3** and **5**, and the results discussed in the main text were obtained by the restricted closed-shell calculations. More importantly, the restricted calculations also successfully reproduced the Mössbauer parameters within the uncertainty of the computations.

Table S2. Computed key metric parameters of complex **3**.

	Fe-P (Å)	Fe-C (Å)	Fe-P-Fe (˚)
BP86 (UKS = RKS)	1.998, 1.993	2.007, 1.952, 2.021, 1.960, 2.006, 1.952, 2.009, 1.951	178.2
TPSSh (UKS = RKS)	1.968, 1.968	2.017, 1.956, 2.015, 1.953, 2.017, 1.955, 2.015, 1.954	178.6
RKS B3LYP	1.969, 1.971	2.040, 1.964, 2.059, 1.993, 2.043, 1.987, 2.046, 1.967	170.7
UKS B3LYP BS(3,3)	2.121, 2.115	2.061, 2.023, 2.056, 2.001, 2.040, 2.010, 2.057, 1.994	159.3
Exp.	1.993, 1.998	2.021, 1.960, 2.007, 1.952, 2.008, 1.951, 2.006, 1.952	178.3

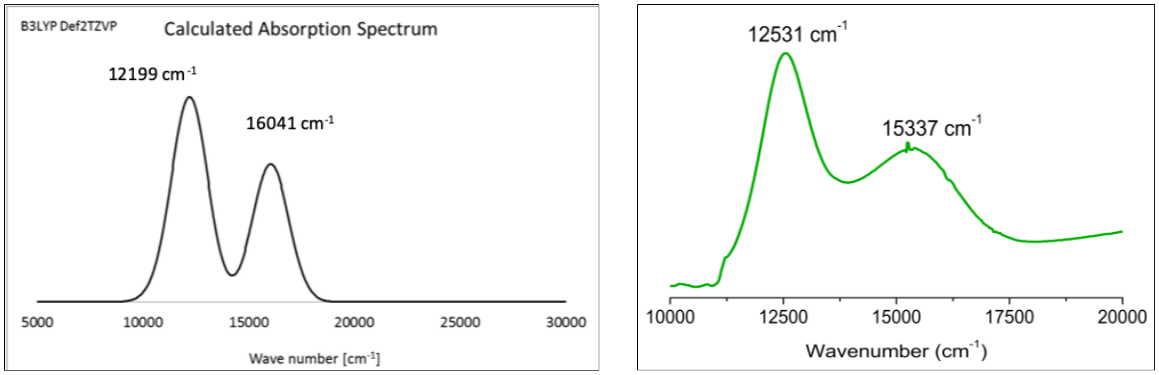

Figure S61. Computed (left) and experimental (right) absorption spectrum of complex **3**.

Table S3. Computed electronic transitions.

	Assignment	Oscillator strength
11053 cm^{-1} (905 nm)	nb $\sigma \rightarrow \pi^*$	0.0019
11107 cm^{-1} (900 nm)	nb $\sigma \rightarrow \pi^*$	0.0018
12223 cm^{-1} (818 nm)	nb $\sigma \rightarrow \sigma^*$	0.1224
16032 cm^{-1} (624 nm)	nb $\pi \rightarrow \sigma^*$	0.0419
16062 cm^{-1} (623 nm)	nb $\pi \rightarrow \sigma^*$	0.0415

As shown in Figure S53, because of the bent Fe-P-Fe core, the three non-bonding orbitals (*vide infra*) involving the Fe-d_{z2}, -d_{xz} and -d_{yz} orbitals of each iron center in the spin-up manifold are slightly different from those in the spin-down set. Thus, the B3LYP broken symmetry calculations in fact deliver a similar bonding picture as those predicted by BP86 and TPSSh.

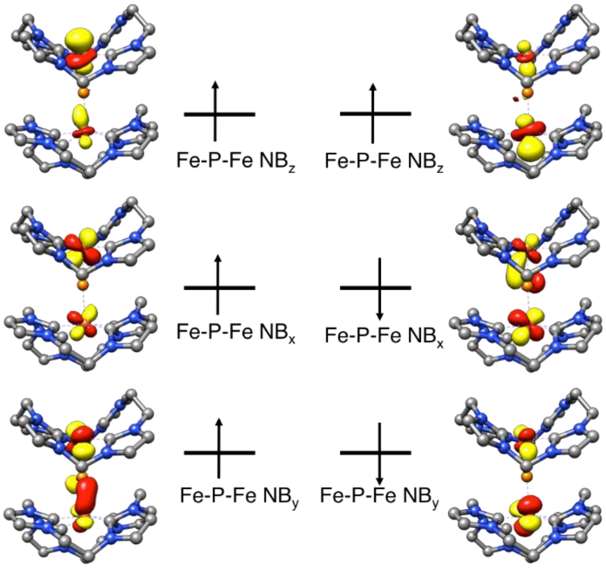

Figure S62. Molecular diagram for Complex **3**

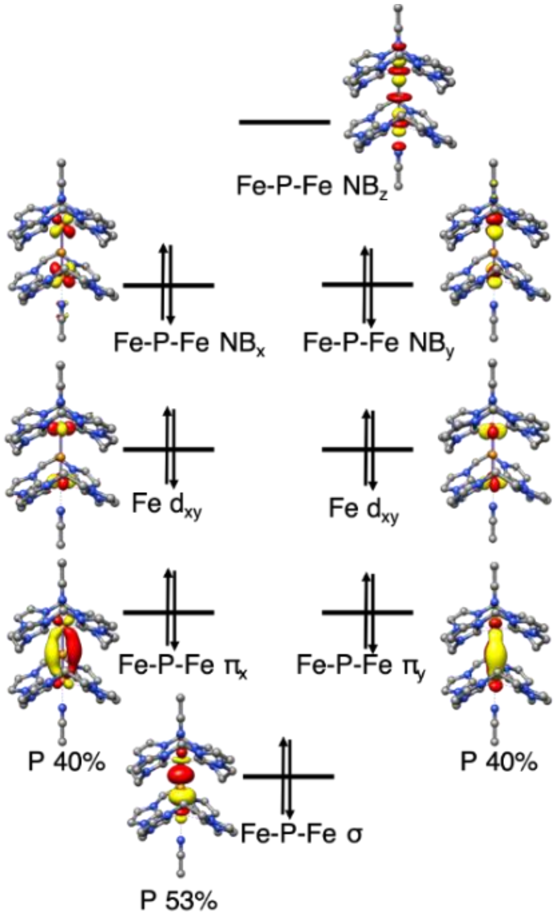

Figure S63. Molecular orbital diagram of complex **5.**

14. References

[1] S. Meyer, I. Klawitter, S. Demeshko, E. Bill, F. Meyer, *Angew. Chem. In. Ed.* **2013**, *52*, 901-905

[2] F. F. Puschmann, D. Stein, D. Heift, C. Hendriksen, Z. A. Gal, H. F. Grützmacher, H. Grützmacher, *Angew. Chem. Int. Ed.* **2011**, *50*, 8420 -8423

[3] H. E. Gottlieb, V. Kotlyar, A. Nudelman, *J. Org. Chem.* **1997**, *62*, 7512-7515

[4] a) G. R. Hanson, K. E. Gates, C. J. Noble, M. Griffin, A. Mitchell, S. Benson, *J. Inorg. Biochem*. **2004**, *98*, 903-916; b) S. Stoll, A. Schweiger, *J. Magn. Reson*. **2006**, *178*, 42-55

[5] E. Bill, Max-Planck Institute for Chemical Energy Conversion, Mülheim/Ruhr, Germany.

[6] E. Bill, *julX, Program for Simulation of Molecular Magnetic Data*, Max-Planck Institute for Chemical Energy Conversion, Mülheim/Ruhr, **2008**.

[7] a) G. M. Sheldrick, *Acta Cryst*. **2015**, *A71*, 3-8; b) G. M. Sheldrick, *Acta Cryst.* **2015**, *C71*, 3-8.

[8] *X-RED*; STOE & CIE GmbH, Darmstadt, Germany, 2002.

[9] (a) A. D. Becke, *J. Chem. Phys.* **1993**, *98*, 5648-5652. (b) C. T. Lee, W. T. Yang, R. G. Parr, *Phys. Rev. B* **1988**, *37*, 785-789

[10] A. Schäfer, C. Huber, R. Ahlrichs, *J. Chem. Phys.* **1994**, *100*, 5829-5835

[11] A. Schäfer, C. Huber, R. Ahlrichs, *J. Chem. Phys.* **1992**, *97*, 2571-2577

[12] K. Eichkorn, O.Treutler, H. Öhm, M. Häser, R. Ahlrichs, *Chem. Phys. Lett.* **1995**, *240*, 283-290

[13] K. Eichkorn, O.Treutler, H. Öhm, M. Häser, R. Ahlrichs, *Chem. Phys. Lett.* **1995**, *242*, 652-660

[14] F. Neese, F.Wennmohs, A. Hansen, U. Becker, *Chem. Phys.* **2009**, *356*, 98-109

[15] F. Neese, *Inorg. Chim. Acta* **2002**, *337*, 181-192

[16] M. Römelt, S. Ye, F. Neese, *Inorg. Chem.* **2009**, *48*, 784-785

[17] S. Sinnecker, L. D. Slep, E. Bill, F. Neese, *Inorg. Chem.* **2005,** *44*, 2245-2254

[18] F. Neese, *WIREs Comput. Mol. Sci.* **2018**, *8*:e1327
